# A new dimension of simplified science communication: the easiness effect of science popularization in animated video abstracts

**DOI:** 10.3389/fpsyg.2025.1584695

**Published:** 2025-07-02

**Authors:** Sara Salzmann, Charlotte Walther, Kai Kaspar

**Affiliations:** Department of Psychology, University of Cologne, Cologne, Germany

**Keywords:** science communication, science popularization, video abstracts, easiness effect, debiasing intervention, social media reactions, knowledge-enhancing reactions, cognitive bias

## Abstract

**Introduction:**

A common approach to make scientific information more accessible for the broader public, is making it easier to understand and translating it into more appealing formats, like short and entertaining online videos. However, simplifying scientific content can have negative impact on consumers, as it can lead to the so-called easiness effect, a cognitive bias which can include an overestimation of one’s own competencies. In the context of scientific studies, this bias has previously only been demonstrated by comparing text-based scientific abstracts with easier-to-understand plain language summaries (PLS). With several unsuccessful approaches in research to reduce the easiness effect, a promising new method might be using debiasing videos as they have been shown to reduce cognitive biases in other contexts. The present study expands the research by exploring the easiness effect in animated video abstracts and investigates whether a debiasing video can reduce it.

**Method:**

This experiment realized a 2 (video abstract type: PLS versus scientific abstracts) × 2 (debiasing video: shown versus not shown) between-participants design. Overall, 179 participants received four abstracts and rated (1) study comprehensibility, (2) perceived study credibility, (3) confidence in one’s ability to evaluate the study, and (4) perceived ability to make decisions without further information. Also, intended consumer reactions (knowledge-enhancing and social media reactions) were collected.

**Results:**

Animated PLS, compared to animated scientific abstracts, actually enhanced comprehensibility of scientific content. This effect was accompanied by a significant easiness effect, as PLS were perceived as more credible and they produced a higher confidence in the recipients’ perceived ability to evaluate the study. No differences in consumer reactions were observed between abstract types. Also, the video-based debiasing intervention did not affect study evaluation.

**Discussion:**

The easiness effect can be reliably generated in video abstracts and it is very robust, as it persists even if a debiasing intervention is carried out beforehand. This study underscores the need for responsible communication strategies in science popularization and shifts the focus to the increasingly popular video abstracts. The results provide a valuable starting point for further research on how video-based science communication can be optimized to convey scientific information effectively.

## 1 Introduction

The internet has become the leading source for information and a key platform for acquiring scientific knowledge (Takahashi and Tandoc, 2016; Cinelli et al., 2020). In this context, effectively communicating scientific content to non-experts has become increasingly important. Specifically, during the COVID-19 pandemic, scientists played an essential role in disseminating information (Fraser et al., 2021). The term “infodemic” emphasized the accompanying overwhelming spread of information, which highlights the challenges in communicating scientific findings in clear and actionable ways (Eysenbach, 2020). The overwhelming spread of content is particularly evident on social media, where many—especially young adults—turn to information. Young adults are among the most active social media users and rely heavily on these platforms as their primary source for science-related content (Hargittai et al., 2018). Platforms like YouTube play a central role in engaging the public with science due to their accessibility, enjoyment, and ease of use (Rosenthal, 2018). In fact, video-based formats are one of the dominant ways of imparting knowledge and platforms such as YouTube are increasingly cultivating these forms of information presentation and consumption in many subject areas such as politics (Zimmermann et al., 2020), health (Osman et al., 2022), and education (Shoufan and Mohamed, 2022).

In contrast to the large amount of simplified content online, specialized articles in scientific journals provide complex, rigorous, and well-founded insights. However, these are primarily consumed and reviewed by experts within the scientific community and rarely reach the broader public (Laine et al., 2007). These experts must have engaged in specialized study, completed extensive training, and accumulated relevant experience to be capable of understanding complex and specific problems in their scientific field (Thomm and Bromme, 2011). In contrast, the broader public consisting of laypeople—including well-educated laypeople with a general academic background—lacks such specialized expertise and remains distinct from experts when dealing with topics outside their own domain of expertise (Scharrer et al., 2013). Taken together, this raises the challenge of how scientific content can be communicated in a way that the broader public can understand, while still maintaining scientific accuracy. In recent years, scientists tried different approaches to address this challenge. A practical and increasingly popular solution is the use of plain language summaries (PLS), which simplify the core findings of scientific research into laypeople-friendly formats without compromising on accuracy (Stoll et al., 2022). Additionally, innovative formats such as comics (Farinella, 2018) and storytelling (Joubert et al., 2019) have been employed to make scientific findings not only more comprehensible but also engaging and appealing to a wider audience. In particular, given the important role of video-based information presentation, more and more efforts are being made to present the content of scientific studies in such video formats, thus taking into account current media usage behavior (cf. Bonnevie et al., 2023; Ferreira et al., 2021; Liu, 2022).

However, the complexity of information presentation plays a decisive role in the attempt to make science communication simpler and clearer. While overly complex content can be difficult for the broader public, over-simplification of scientific information can also lead to unintended negative effects. One such effect is the *easiness effect of science popularization* (hereinafter referred to as the *easiness effect*), a cognitive bias suggesting that comprehensible information is perceived as more credible than less comprehensible information, leading to greater acceptance of the presented claims (Scharrer et al., 2017; Kerwer et al., 2021). Effective science communication must therefore strike a balance between accessibility and accuracy, avoiding both over-complexification and over-simplification of scientific content. The present study hence aims to explore the use of animated video abstracts to present scientific content in two modes (simplified versus non-simplified), focusing on whether the cognitive bias known as the easiness effect occurs in these formats. Additionally, it examines the effectiveness of a video-based debiasing intervention as a strategy to reduce this potential easiness effect.

### 1.1 Easiness effect of science popularization

Simplifying scientific content is an effective way to enhance its comprehensibility, accessibility, and appeal to broader audiences. However, simplification also carries potential risks, including the easiness effect (e.g., Scharrer et al., 2017)—a cognitive bias, which leads to simplified information appearing more credible than it objectively is and which increases trust in one’s own judgment while also reducing the desire to cross-evaluate an information, e.g., by consulting an expert. Over the past decade, a few studies have examined the easiness effect, differing slightly in the operationalizations which were applied but consistently focusing on three key facets: First, several studies showed that non-experts rated more comprehensible texts as more credible compared to less comprehensible texts (Scharrer et al., 2012, 2013, 2014, 2019, 2021, 2022; Kerwer et al., 2021). Second, participants were also more confident about making a decision on their own based on more comprehensible texts compared to less comprehensible texts. In particular, this included either a hypothetical but realistic scenario in which a decision about the accuracy of a scientific claim needed to be made (Scharrer et al., 2012, 2013, 2014, 2017, 2019, 2021, 2022) or a metacognitive assessment of one’s own competence in evaluating scientific claims on one’s own (Mohajerzad et al., 2024; Kerwer et al., 2021). Third, studies also either showed lower ratings of non-experts’ desire to consult with an expert for making a judgment (Scharrer et al., 2012, 2013, 2014, 2017, 2021; Mohajerzad et al., 2024) or higher confidence in decision-making without consulting an expert (Kerwer et al., 2021) based on more comprehensible texts compared to less comprehensible texts.

The easiness effect has been shown to be relatively robust. It even occurred—albeit partially less strongly—when the simplified information was presented in a controversial way (Scharrer et al., 2013), described as complex (Scharrer et al., 2014), came from a non-credible source (Scharrer et al., 2019), or was framed with a warning message regarding its content (Scharrer et al., 2022). The easiness effect also persists for information across disciplines including medicine (Scharrer et al., 2012, 2013, 2014, 2017, 2019, 2022), climate policy (Scharrer et al., 2012, 2021), technology (Bullock et al., 2019), social psychology (Kerwer et al., 2021), and educational research (Mohajerzad et al., 2024). Additionally, previous studies successfully demonstrated the easiness effect in samples with different types of participants including well-educated laypeople, more precisely, students (Scharrer et al., 2013, 2014, 2019, 2021; Kerwer et al., 2021; Thon and Jucks, 2017), the broader public (Scharrer et al., 2017, 2022; Bullock et al., 2019), as well as professional practitioners (Mohajerzad et al., 2024). Lastly, the easiness effect was found in studies using varying stimulus material including researcher-generated texts with fictional (Scharrer et al., 2012, 2013, 2014, 2019, 2021), false yet plausible (Scharrer et al., 2022), and accurate scientific claims (Bullock et al., 2019; Mohajerzad et al., 2024), as well as articles from real-life popular and expert science magazines (Scharrer et al., 2017) and real-life scientific abstracts (Kerwer et al., 2021).

There are two potential mechanisms which could explain the occurrence of the easiness effect. The first is a misjudgment of complexity: when laypeople easily comprehend simplified scientific content, they may conclude that the entire underlying scientific construct must be correspondingly simple (Goldman and Bisanz, 2002; Scharrer et al., 2017). Laypeople’s misjudgment of the subject’s complexity can then lead to an overestimation of their ability to evaluate the provided information appropriately. The second mechanism is based on fluency processing, which describes the subjective ease experienced during cognitive tasks (Hansen et al., 2008; Scharrer et al., 2017, p. 1006). When a mental task is easy—like reading a comprehensible text—it can be processed fast and effortless so that the information presented is therefore perceived as more familiar and positive (e.g., Bullock et al., 2019). This can result in a more pronounced evaluation of the information as true (Hansen et al., 2008; Reber and Schwarz, 1999) and in a stronger perceived knowledge about the topic and higher confidence in evaluating the information (Kerwer et al., 2021; Scharrer et al., 2017). In both cases, when information is easy to comprehend, laypeople tend to feel more confident in their ability to evaluate it. However, this can lead to an overestimation of their own knowledge and a greater vulnerability to misinformation (Scharrer et al., 2012, 2017).

Interestingly, the easiness effect is not universal. For example, Scharrer et al. (2021) also manipulated belief consistency of the presented information and showed that the easiness effect only occurred when the presented information was consistent with the participants’ prior beliefs. Additionally—and contrary to the predictions of the easiness effect—Thomm and Bromme (2011) observed that texts with scientific features (e.g., references, methodological details, active and passive language) were rated not only as more scientific but also as more credible, compared to texts without scientific features. The higher credibility can be explained by the so called “scientificness effect” (Thomm and Bromme, 2011, p. 187). While the easiness effect predicts a higher credibility for easier information, the scientificness effect assumes that more difficult scientific information leads to a higher credibility. Some studies supported these findings (e.g., Thomm and Bromme, 2011; Bromme et al., 2015), whereas other studies—in line with the easiness effect—found a higher credibility for easier texts (e.g., Scharrer et al., 2013; Kerwer et al., 2021).

As established above, when scientific content is simplified to make it more accessible to the broader public, the easiness effect can emerge. Considering its consequences, there might be potential benefits for science communication and individuals, for example a higher credibility of relevant scientific information within the broader public or increased confidence in decision-making which might foster science-based decisions. The prerequisite for these aspects to be considered positive is that the underlying scientific information is valid and the applied simplification accurate. While higher perceived credibility and confidence in decision-making might also have positive outcomes, the reduced willingness to consult an expert or to obtain additional information plays a more critical role since it generally conflicts with fundamental principles of scientific practice and leads to a lacking cross-evaluation of potentially wrong information. In general, the easiness effect and its consequences become problematic when simplification of scientific information results in an unjustified increase of perceived credibility—especially in case of false information or inaccurate simplification—or in recipients overestimating their own competence. This can lead to harmful real-life implications, for example, individuals might decide to follow questionable health trends, which were comprehensibly and convincingly presented, but are scientifically invalid. Hence, to mitigate potential negative outcomes, a deeper understanding of the easiness effect and its characteristics is essential, alongside strategies to reduce its occurrence. To enable a thorough examination of the easiness effect, it is crucial to conceptualize and standardize the simplification process. Standardized text material which presents scientific findings in an academic yet easily comprehensible way may be a promising stimulus for addressing both standardization and ecological validity.

### 1.2 Plain language summaries

In order to make texts with scientific content more understandable for laypeople, researchers sometimes translated technical language and excluded difficult information (Scharrer et al., 2013), added jargon terms (Bullock et al., 2019), or used articles from popularized magazines (Scharrer et al., 2017). However, the resulting formats may differ considerably between studies. Hence, the format of the plain language summary (PLS) was invented to standardize such scientific texts that are intended to be easier to understand for laypeople. While scientific abstracts are intended for expert audiences, PLS provide non-technical explanations of the study’s rationale, methods, and findings, allowing non-experts to correctly interpret scientific information (Stricker et al., 2020). The Cochrane Collaboration (Pitcher et al., 2022) established a framework with diverse PLS guidelines, ensuring standardized simplification of scientific information across research fields by defining length, statistical methods, and reporting of quality of evidence. PLS therefore have a similar length as scientific abstracts, are written by the authors themselves (Fitz Gibbon et al., 2020), prioritize theoretical derivation and practical use while avoiding scientific jargon (Hauck, 2019; Pitcher et al., 2022), offer more transparency (Barnes and Patrick, 2019; Kuehne and Olden, 2015), and are reported to be easier to comprehend than traditional scientific summaries (Buljan et al., 2018; Fitz Gibbon et al., 2020; Santesso et al., 2015). Recent research by Stricker et al. (2020) examined 103 PLS and their corresponding scientific abstracts in social and political psychology, indicating that PLS were easier to read than scientific abstracts. Kerwer et al. (2021) supported the existence of the easiness effect by comparing PLS and scientific abstracts from the study by Stricker et al. (2020), revealing that in comparison to scientific abstracts, PLS were rated as more comprehensible and credible, and led to increased confidence in decision-making without consulting an expert as well as higher interest in accessing the full study. This provided evidence of the easiness effect in standardized scientific research summaries within the psychological research field. As a practical approach, PLS provide a standardized method to make scientific information more accessible while also offering a methodical tool to investigate the easiness effect in simplified content.

### 1.3 Transfer to animated video abstracts

The easiness effect has only been studied in the context of textual information so far but given the popularity and widespread use of video-based content, there is growing interest in exploring more engaging and illustrative formats for communicating scientific content (Rosenthal, 2018). Visual elements such as images, illustrations, and infographics offer several advantages (Levie and Lentz, 1982; Mayer, 1989; Mayer and Gallini, 1990). For example, Buljan et al. (2018) found that infographics were rated as more user-friendly and provided a better reading experience compared to scientific abstracts and even PLS.

The Cognitive Theory of Multimedia Learning (Mayer, 2001, 2005) provides a framework for understanding the advantages of illustrative formats, especially animations, and guidelines for their effective application. The Cognitive Theory of Multimedia Learning is based on three assumptions: the dual-channel assumption states that verbal and pictorial information are processed in two independent but interacting systems; the limited capacity assumption states that these channels have restricted capacity, requiring selective information processing in the working memory; the active learning assumption states that learning is an active process where learners construct knowledge in a meaningful manner (Mayer, 2001, 2005; Sorden, 2012). Additionally, Mayer (2014) presents principles to optimize processing and enhance effective learning in multimedia environments. Among these principles, the multimedia principle is particularly relevant. It suggests that students learn more effectively when verbal and pictorial information are presented together rather than text alone, activating both channels and thus leading to better mental connections and deeper learning. Although the Cognitive Theory of Multimedia Learning specifically focuses on learning processes, its principles are generally relevant for the cognitive processing of scientific content, which is the subject of the present study.

Building on the effectiveness of static visuals, animations are increasingly used to convey scientific content. Animations use dynamic graphical elements to represent complex phenomena in a visually engaging and illustrative manner, potentially enhancing the comprehensibility of scientific explanations (Glaser and Schwan, 2015; Mayer and Anderson, 1992; Mayer and Moreno, 2002). In the context of PLS, Bredbenner and Simon (2019) compared different presentation formats of scientific summaries (including video abstracts that were created using a “whiteboard explainer style,” p. 3) with visuals drawn and synchronized to narration. They found that video abstracts (and text-based PLS) were significantly more effective than both graphical abstracts and published abstracts in enhancing comprehension, perceived understanding, enjoyment, and the desire for further updates. This effect was consistent across participants from scientific, science-related, and non-science careers, suggesting that these formats are universally beneficial for communicating scientific findings.

In summary, animated PLS may represent a promising tool in science communication, combining the benefits of visuals with dynamic and engaging features and the standardized approach of PLS. However, their potential to enhance ease of understanding could also further amplify the easiness effect—a phenomenon that remains unexamined in this specific context. The present study therefore addresses this research gap by investigating the easiness effect through a direct comparison of animated PLS versus animated scientific abstracts (i.e., *video abstract type*). To achieve this, three hypotheses were formulated, each addressing one facet of the easiness effect. Following the operationalization by Kerwer et al. (2021), the easiness effect is characterized by a combination of (1) perceived study credibility, (2) confidence in one’s ability to evaluate the study, and (3) perceived ability to make decisions without further information:

*H1a*: Participants who receive animated PLS report higher perceived study credibility compared to participants who receive animated scientific abstracts that are not tailored to laypeople.

*H1b*: Participants who receive animated PLS report higher confidence in their ability to evaluate the study compared to participants who receive animated scientific abstracts that are not tailored to laypeople.

*H1c*: Participants who receive animated PLS report a higher ability to make decisions without further information compared to participants who receive animated scientific abstracts that are not tailored to laypeople.

### 1.4 Reducing the easiness effect: debiasing interventions

When information is presented in an easily comprehensible manner, laypeople not only tend to evaluate the information as more credible, they also tend to overestimate their competence and may indicate that they do not need further cross-checking before making decisions (e.g., Scharrer et al., 2012, 2013, 2014). This tendency underscores the need for effective strategies to reduce the easiness effect. Recent research has explored various strategies to reduce this effect. For example, Scharrer et al. (2019) manipulated source credibility but found that the effect persisted, even when information was described as less credible. Another strategy involved framing the topic as controversial (Scharrer et al., 2013); while some aspects of the easiness effect were reduced (e.g., claim agreement, trust in one’s own decision), other aspects remained unaffected (e.g., perceived credibility, desire to consult an expert). Similarly, explicit information about the topic’s complexity only partially reduced the effect (Scharrer et al., 2014). In another study, Scharrer et al. (2022) examined how warning labels influence laypeople’s evaluation of simplified scientific misinformation. The warning labels contained the message that independent fact-checkers dispute the presented content. Presenting these warning labels effectively enhanced laypeople’s skepticism toward scientific misinformation as participants who read texts with warning labels showed significantly lower agreement with the claims and rated the texts as less credible. Furthermore, warning labels led to a stronger desire to consult experts, indicating reduced confidence in one’s own judgment. However, the easiness effect persisted despite the presence of warning labels. Easy-to-understand texts were perceived as more persuasive than complex ones, regardless of the presence of warning labels. This suggests that warning labels cannot completely counteract the persuasive advantage of simply presented scientific content, particularly in the context of misinformation. These findings highlight the robustness of the easiness effect and the difficulty of fully reducing its impact.

Another line of research, however, has demonstrated the potential of debiasing videos to effectively reduce cognitive biases. For example, Dunbar et al. (2014) presented participants debiasing videos of different lengths (30 versus 60 min) either once or twice to reduce the confirmation bias and the fundamental attribution error. The videos consisted of five vignettes with realistic scenarios, in which the protagonists reveal a specific cognitive bias, and a moderating host explains the biases and presents specific mitigation strategies. Results showed that the debiasing videos improved knowledge of and familiarity with the biases, with the longer videos being more effective over time in enhancing knowledge, and the double exposure leading to greater familiarity. Similarly, Rhodes et al. (2017) showed that a debiasing video of 30–35 min length including real-life vignettes and a scientist who explains cognitive biases (e. g., confirmation bias, fundamental attribution error) significantly improved declarative knowledge about the biases, with effects lasting over a period of 8–12 weeks. Morewedge et al. (2015) also found that a one-shot debiasing training intervention—more specifically, watching a 30-min video explaining heuristics in general, defining specific biases, presenting vignettes which demonstrate the biases, giving additional examples, suggesting reduction strategies, and concluding with a 2-min review of the content—effectively reduced cognitive biases, such as the fundamental attribution error and confirmation bias, with medium to large effects persisting for several months. Likewise, Rusmana et al. (2020) achieved a significant reduction of the overconfidence bias through a debiasing video with a small effect size. The video used by Rusmana et al. (2020) followed a similar structure to that of Morewedge et al. (2015) by defining and explaining the bias, discussing related biases, highlighting negative consequences, and providing strategies to overcome it. While research on debiasing has focused on countering other cognitive biases, to the best of our knowledge, no research has specifically addressed debiasing interventions to reduce the easiness effect in science communication.

In general, approaches to reduce cognitive biases can be broadly categorized into three groups: One approach involves changing incentives, for example by rewarding desired behavior or penalizing non-desired behavior. A second approach involves modifying the presentation of the information, as seen in previous (non-video) attempts to reduce the easiness effect (e.g., Scharrer et al., 2013, 2014, 2019, 2022), which used warning messages or highlighted the complexity of information. A third approach focuses on debiasing videos—such as those used by Dunbar et al. (2014), Morewedge et al. (2015), Rhodes et al. (2017), and Rusmana et al. (2020)—that try to reduce cognitive biases by improving decision-making ability through training. This approach refers to the two-system model of reasoning (Milkman et al., 2009). This model suggests that decision making involves two systems (Evans, 2003; Kahneman, 2003; Morewedge and Kahneman, 2010), whereby system 1 is for intuitive thinking (unconscious, fast, and effortless) and system 2 for reflective thinking (conscious, slow, and effortful). Biases often arise from system 1 thinking due to undervaluing important information and fallibility (Kahneman, 2003). A debiasing video aims to activate system 2 to encourage effortful and conscious thinking to prevent cognitive biases.

Against this background, the present study aims to reduce the easiness effect by using an animated debiasing video specifically produced for the purpose of the present study on video abstracts and designed to activate system 2, promoting reflective thinking and reducing the influence of cognitive biases (cf. Rusmana et al., 2020). The central research question is whether such a debiasing video can significantly reduce the easiness effect. The corresponding hypotheses are as follows:

*H2a*: Participants who receive a debiasing video prior to the video abstracts (PLS or scientific abstracts) report lower perceived study credibility compared to participants who do not receive a debiasing video.

*H2b*: Participants who receive a debiasing video prior to the video abstracts report lower confidence in their ability to evaluate the study compared to participants who do not receive a debiasing video.

*H2c*: Participants who receive a debiasing video prior to the video abstracts report a lower ability to make decisions without further information compared to participants who do not receive a debiasing video.

### 1.5 Interaction between video abstract type and debiasing video

Additionally, the study explores the interaction between the presented type of video abstract (PLS versus scientific abstracts) and the debiasing video. If individuals viewing animated PLS tend to overestimate themselves more than those viewing animated scientific abstracts, it is hypothesized that the debiasing video will have a stronger effect on reducing overestimation when presented with animated PLS. The hypotheses are formulated as follows:

*H3a*: For participants receiving animated PLS, perceived study credibility is reduced more by a debiasing video compared to participants receiving animated scientific abstracts.

*H3b*: For participants receiving animated PLS, confidence in one’s ability to evaluate the study is reduced more by a debiasing video compared to participants receiving animated scientific abstracts.

*H3c*: For participants receiving animated PLS, the reported ability to make decisions without further information is reduced more by a debiasing video compared to participants receiving animated scientific abstracts.

### 1.6 Impact of video abstract type on consumer reactions to video abstracts

The complexity of presented information influences how individuals perceive and engage with it (Kerwer et al., 2021; Shoufan, 2019; Möller et al., 2019). To further conceptualize audience engagement, Shao (2009) proposed a framework based on the Uses and Gratifications Theory (Katz et al., 1973) of how individuals engage with media. The central claim of the Uses and Gratifications Theory is that individuals actively and purposefully engage with media to fulfill their specific needs. According to this framework, McQuail (1987) postulated core motivations for media use—information, entertainment, social interaction, and self-expression—and that these can be reflected in three interdependent activities: (1) consumption, including viewing content for information and entertainment (fulfilling the need for information and entertainment), (2) participation, including liking, sharing, and commenting to enhance social connections (fulfilling the need for social interaction), and (3) content production, including the creation of media content (fulfilling the need for self-expression and self-actualization). Building on this, Khan (2017) introduced the concept of behavioral engagement to examine further consumption and participation on YouTube videos. By investigating reactions to YouTube videos and focusing on consumption and participation in relation to motives, the study revealed that when participants seek information, they both participate by liking and consume by viewing and reading comments.

While limited research has focused on consumer reactions to simplified and complex content so far, some studies provide valuable insights. For example, Shoufan (2019) found that content comprehensibility predicts the number of likes for educational YouTube videos. Similarly, Möller et al. (2019) found that entertaining videos received more likes and comments compared to difficult political videos, with dislikes not significantly differing between the two types of videos. These findings suggest that easier content may promote enjoyment and *social media reactions*.

Regarding reactions aimed at increasing the recipient’s individual knowledge (*knowledge-enhancing reactions*), Kerwer et al. (2021) found that participants who received PLS showed a higher demand to seek the full-text article compared to participants who received scientific abstracts. This contradicts the easiness effect assumption, which states that simplified content should reduce the demand for further information, whereas a scientific abstract—due to difficulties in understanding scientific terminology—should enhance the demand for further information. One explanation for this result could be that easier content is perceived as more enjoyable, with reduced mental effort linked to positive affect (Winkielman and Cacioppo, 2001). While these findings align with prior research suggesting that making science more enjoyable and interesting results in greater engagement (Falk et al., 2016), the easiness effect suggests that presenting PLS may lead to lower knowledge-enhancing reactions (e.g., seeking full-text article) compared to scientific abstracts. Research by Kaspar et al. (2015) found that scientific abstracts presented in serif fonts were associated with higher perceived clarity and scientific quality, significantly increasing interest in reading the full-text compared to sans-serif fonts. This effect occurred despite the lower reading speed of abstracts in serif fonts (i.e., higher cognitive demands), which contrasts with the higher processing fluency associated with sans-serif fonts. Taken together these mixed findings, receiving PLS could result in an increased demand to seek the full-text article due to better comprehension and heightened interest, but it could also result in decreased demand due to perceived triviality. These mixed findings highlight the need for the present study to not only re-evaluate the exploratory findings of Kerwer et al. (2021) but also to investigate consumer reactions in more depth. Therefore, the hypotheses are as follows:

*H4a*: Participants who receive animated PLS differ in their intended knowledge-enhancing reactions (reading comments related to the video abstract, watching another video of the research summary, watching another video of the same topic, getting the full access to the corresponding paper) from participants who receive animated scientific abstracts.

*H4b*: Participants who receive animated PLS differ in their intended social media reactions (liking, disliking, sharing, and commenting) from participants who receive animated scientific abstracts.

### 1.7 Impact of debiasing video on consumer reactions to video abstracts

The debiasing video is expected to activate system 2 thinking (see section 1.4), characterized by slow and effortful processing. Therefore, it is hypothesized that the debiasing video will incentivize participants to seek additional information and increase knowledge-enhancing reactions. Additionally, the extent of social media reactions may be affected by increased examination of the video. Participants may engage in more thoughtful reactions, being cautious in their responses, or their interest might intensify, leading to stronger reactions. Since sufficient research on this matter is lacking, the study will explore these questions in an exploratory manner:

*RQ1*: How do participants, who receive a debiasing video prior to the video abstracts, differ in their reactions to the presented video abstracts, specifically in terms of intended knowledge-enhancing reactions and social media reactions?

## 2 Method

### 2.1 Participants

An *a priori* power analysis was computed using GPower 3.1.9.7 software (Faul et al., 2009). Aiming at a medium-sized effect of *f* = 0.25 (cf. Bullock et al., 2019; Scharrer et al., 2013, 2014, 2019; Thon and Jucks, 2017), a desired power of 0.8 in the ANOVA (fixed effects, special, main effects and interactions) and α = 0.05, the required minimum sample size was *n* = 128. A convenience sample that met the inclusion criteria (see below) was recruited through messaging apps (WhatsApp), social media (Facebook, Instagram), the SurveyCircle website (SurveyCircle, 2016), and the University of Cologne’s website and courses. Participants were asked to complete an online experiment, and it was ensured they understood the task and provided honest responses. Data collection took 5 months.

A total of 369 participants initially took part in the study. A substantial part was excluded due to incomplete questionnaires (*n* = 105), skipping videos (*n* = 12), or not meeting the pre-selection criteria (*n* = 65), although these were communicated transparently. The pre-selection criteria required participants not to be younger than 18 years, currently enrolled at a university with a study program in German language, and not to be studying psychology or a similar subject (e.g., economic psychology) to capture a subgroup of well-educated laypeople. This particular subgroup was targeted due to two reasons. First, we selected persons currently enrolled at a university because young, educated individuals search for scientific information online most frequently (Andreassen et al., 2007). Also, this group can be assumed to have general scientific literacy and interest. Second, we excluded persons studying psychology (and similar topics) because we expect them to have a deeper understanding of the exact subject matter which our stimulus material was focused on, as we used psychological study abstracts. Including experts would not have been in line with the present investigation of the easiness effect and prior knowledge of the subject matter would have impacted the responses. One participant was under the age of 18, and a further 64 participants were excluded because they were studying psychology or related subjects. Additionally, eight participants with processing times being three standard deviations above the mean were excluded to ensure the debiasing video’s effect was not influenced by extended processing time or extended breaks.

The final sample therefore included 179 participants, consisting of 135 women, 43 men, and one diverse person. Participants’ ages ranged from 18 to 45 years, with a mean age of 25.10 years (*SD* = 3.72). The majority pursued their bachelor’s (40.8%) or master’s (53.1%) degree, an additional 2.9% of the sample was currently working on their PhD, and 1.7% on a state exam. The highest level of education varied, with 31.8% holding a high-school diploma, 5.6% having completed vocational training, 55.3% having a bachelor’s degree, and 3.4% a master’s degree. On average, participants were in the 8.67th semester (*SD* = 4.48) and were studying various subjects, such as business administration, architecture, and social science.

### 2.2 Study design

An experimental 2 (video abstract type: PLS versus scientific abstracts) × 2 (debiasing video: shown versus not shown) between-participants design was employed to investigate the presence and modulation of the easiness effect in video abstracts. Perceived study credibility (H1a, H2a, H3a), confidence in one’s ability to evaluate the study (H1b, H2b, H3b), perceived ability to make decisions without further information (H1c, H2c, H3c), intended knowledge-enhancing reactions (H4a, RQ1) and intended social media reactions (H4b, RQ1) served as dependent measures.

The distribution of participants across conditions was uneven due to a true randomization process: PLS with debiasing video (*n* = 35), PLS without debiasing video (*n* = 46), scientific abstract with debiasing video (*n* = 42), and scientific abstract without debiasing video (*n* = 56). Participants were not aware of the different conditions or their group assignments.

### 2.3 Materials

An online experiment (including the presentation of video material and subsequent questions) was created and administered using the online survey platform Unipark (Tivian XI GmbH, 2022). Specifically developed videos of the PLS and scientific abstracts were created for the study using the software program VYOND™ (GoAnimate, Inc., 2022) for animation. VYOND™ includes functionalities to create animated videos with customizable scenes and cartoon characters. The corresponding audio tracks were recoded with the software Audacity^®^ (Audacity Team, 2021), an open-source voice recording and audio processing software.

#### 2.3.1 Independent variables

##### 2.3.1.1 Video abstract type

To manipulate the video abstract type, four out of the twelve studies used by Kerwer et al. (2021) were selected based on specific criteria and transformed into animated videos. Kerwer et al. (2021) selected studies from The Journal of Social and Political Psychology for their research due to its inclusion of both scientific abstracts and corresponding PLS versions, its relevance to the broader public, and due to its openly available content under a CC-BY license, making the content suitable for adaptation, reproduction, and distribution. We selected the studies’ abstracts for the present study with animated videos using four main criteria. First, the selected abstracts and corresponding PLS had the least terms difficult to represent visually, for example terms would be considered as difficult because they were lacking common symbolism, were too complex, or there was not an adequate representation in VYOND™ software (e. g., opinion-based identity). Second, since PLSs are usually longer than scientific abstracts, we took into account that the discrepancy in length between the two versions should not be too great. Third, the selected abstracts and PLS covered different topics and, fourth, used different methods (e.g., experimental design, survey, quasi-experimental field study, computer-aided text-analysis). For each study’s abstract, the number of difficult terms (determined by consensus rating from two independent raters), differences in word count between PLS and scientific abstracts, topic, and method were systematically listed and compared. Consequently, the studies of Halmburger et al. (2019), Selvanathan and Lickel (2019), Kende et al. (2017), and Bäck et al. (2018) were found to best meet the established criteria.

The implementation phase involved transforming each study’s scientific abstract and PLS version into video abstracts. The abstracts and PLS were translated to German to ensure participant comprehension of the text. As all participants were enrolled in German-language degree programs, a sufficient level of German language skills was guaranteed. The translated text was then recorded and professionally synchronized with the illustrations during the animation process.

The animation process was guided by principles of multimedia learning (Mayer, 2014) to ensure an effective learning experience with engaging and accessible educational animation videos. Therefore, the animation videos included verbal and pictorial information to enhance learning and to reduce cognitive load in line with the multimedia and the modality principles. Additionally, matching verbal and pictorial information was presented in spatially and temporally close proximity to avoid the need for participants to mentally combine multiple sources of misaligned information (split-attention principle and spatial–temporal contiguity principle). Furthermore, identical information was only presented in one form (with some exceptions due to dramaturgical reasons), but descriptive images were used to improve the understanding of abstract concepts (redundancy principle). Moreover, the animation videos were segmented into learner-friendly portions, with spoken instead of printed text to allow for optimal cognitive capacity to manage essential processing (segmenting principle and modality principle). Apart from that, extraneous processing was reduced by excluding unnecessary material (coherence principle) and adding visual cues to emphasize critical information (signaling principle). Lastly, we employed social cues by using a standard-accented human voice (voice principles) and did not use the speaker’s image on the screen (image principle).

**Figure 1:**
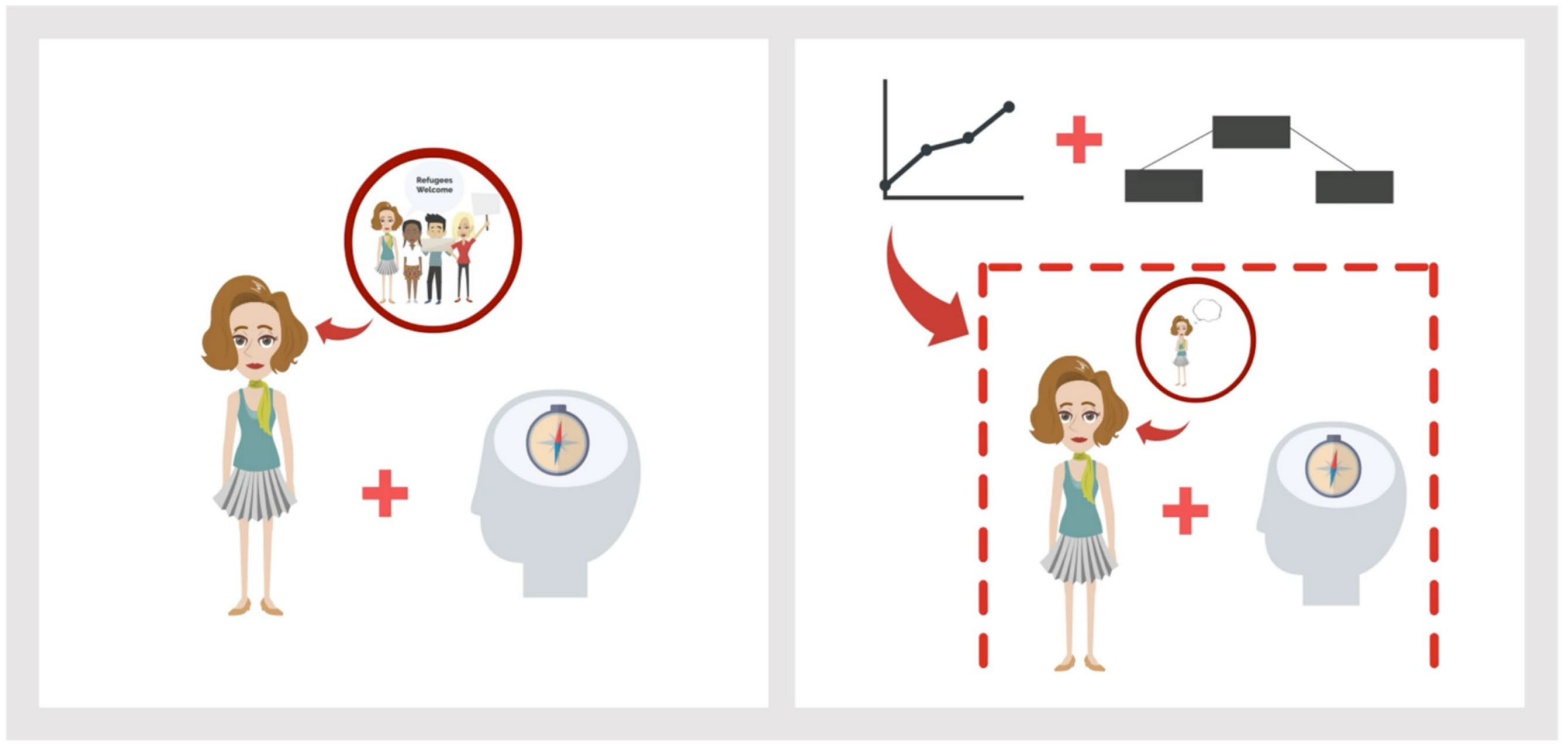
Example frames of the animated PLS (left) and animated scientific abstract (right) of the original text-based abstract created by Kende et al. (2017). The PLS version includes easily comprehensible language and illustrations without statistical terms, while the scientific abstract version uses scientific jargon and presents statistical methods such as hierarchical regression and mediation analysis.

To effectively translate the content of the scientific abstracts and PLS into animations, ideas for representing (complex) terms and concepts were collected by using different methods, including brainstorming, word association exercises, image search, and mind mapping. For instance, the concept of declining trust in politicians was illustrated through images of a handshake, a group of people in suits and a downward-trending arrow. Throughout a multi-stage review process (including the authors and lab staff), all videos were thoroughly assessed and adjusted multiple times to ensure high quality and alignment with the study’s objectives. For each of the four studies, two videos were generated, one for the scientific abstract and one for the PLS version. Compared to the scientific abstract version, the PLS video voice-over includes more comprehensible language (spoken PLS text word for word) and correspondingly less complex visualizations as part of the animation. The scientific abstract video voice-over (spoken abstract text word for word) uses scientific jargon and accordingly more complex visualizations (e. g. of statistical terms). The original abstracts including links to the corresponding publications can be found in Supplementary material A. The average duration per video was about 2 min, with animated PLS being slightly longer. Figure 1 provides an example of the animated content and contrasts the visualizations of an abstract with a PLS version. The videos used for this study are publicly accessible and can be downloaded at OSF (Salzmann, 2025).

##### 2.3.1.2 Debiasing video

In addition to the video abstract type, the debiasing video served as the second independent variable and its creation was based on previous studies that demonstrated the effectiveness of instructional videos in reducing cognitive biases. The script for the debiasing video followed a structure like those used by Rusmana et al. (2020) and Morewedge et al. (2015), starting with an explanation of the easiness effect, followed by its underlying causes, a description of negative effects, and concluding with strategies to counteract the effect. The content was scientifically and empirically grounded but written in an easily comprehensible manner. The animation process was similar to the one used for the video abstracts. The 4.5-min debiasing video was animated, synchronized with audio, and reviewed and adjusted multiple times to ensure high quality. The debiasing video is publicly accessible and can also be downloaded at OSF (Salzmann, 2025). A transcript of the debiasing video in English translation can be found in Supplementary material B.

#### 2.3.2 Dependent variables

The dependent variables were all self-assessed measures. To get a better uniformity for participants and comparability of the measures, all scales were adjusted and ranged from 1 (low) to 7 (high).

##### 2.3.2.1 Manipulation check

Similar to Kerwer et al. (2021), the study assessed whether animated PLS are easier to comprehend than animated scientific abstracts by having participants rate the perceived comprehensibility of the videos (“How do you rate this summary? I find this summary comprehensible.”) using a Likert scale ranging from 1 (*I do not agree at all*) to 7 (*I totally agree*).

##### 2.3.2.2 Easiness effect

Similar to Kerwer et al. (2021), three independent variables were employed to measure the easiness effect. Participants were asked to rate their agreement with three statements using a Likert scales ranging from 1 (*I do not agree at all*) to 7 (*I totally agree*): “I find this summary trustworthy” (perceived study credibility, H1a, H2a, H3a), “Based on this summary, I am able to evaluate the veracity of the corresponding study” (confidence in one’s ability to evaluate the study, H1b, H2b, H3b), and “Based on this summary, I am able to make a decision without needing any further information (e.g., reading the full text or talking to an expert)” (perceived ability to make decisions without further information, H1c, H2c, H3c).

##### 2.3.2.3 Intended consumer reactions

Following Khan’s (2017) concept of examining reactions to YouTube videos, participants were asked to rate their likelihood of seven possible reactions to the video abstracts on a rating scale ranging from 1 (*very unlikely*) to 7 (*very likely*). To measure intended knowledge-enhancing reactions (H4a, RQ1), the following five reactions were collected: reading comments, watching another video of the same subject, watching another video of the same topic, getting full access to the paper in English language, and getting full access to the paper in German language (Cronbach’s α = 0.86). To measure intended social media reactions (H4b, RQ1), the following four reactions were collected: liking, disliking, sharing, and commenting. We subsequently excluded the disliking reaction from the analysis, based on both conceptual and methodological considerations. From a conceptual perspective, practical relevance was reduced as the dislike function on YouTube, as the largest online video portal, is no longer visible for users. Methodologically, factor analyses revealed that loadings on the disliking item were substantially lower compared to the other reactions (see Supplementary Table A) and internal consistency for social media reactions was slightly higher when disliking was excluded (α = 0.77). Given the good internal consistencies, we computed the mean values of intended knowledge-enhancing and social media reactions (without disliking) for further analyses.

##### 2.3.2.4 Covariates

Two self-assessed items, similar to those used by Kerwer et al. (2021), were collected as covariates: participants rated their familiarity with psychological scientific studies (“How familiar are you with psychological scientific studies?”) on a rating scale ranging from 1 (*very low*) to 7 (*very high*) and their general ability to evaluate the veracity of psychological studies (“How would you rate your ability to assess the veracity of psychological scientific studies?”) on a rating scale ranging from 1 (*very low*) to 7 (*very high*).

### 2.4 Procedure

The online experiment took approximately 15 to 25 minutes, depending on the condition. No incentive was announced or given for participation in the study. The study began with a brief introduction and an explanation of its purpose (but without addressing the manipulations in focus), followed by the participants’ agreement to the privacy policy and declaration of consent. Demographic data (including age, gender, current study semester, and section) were collected, along with self-ratings of participants’ familiarity with psychological studies and their general ability to assess the veracity of psychological studies. Subsequently, about half of the participants were assigned to the debiasing condition and viewed the debiasing video explaining the easiness effect, while the other half proceeded directly to the video abstracts. After that, each participant was assigned to either the PLS or scientific abstract condition and watched the four video abstracts of the corresponding type (PLS or scientific abstracts) in randomized order. To prevent premature skipping, the button leading to the next page only appeared 30 seconds after video initiation. All video abstracts (and the debiasing video) were repeatable. After each video abstract, participants rated the perceived comprehensibility (manipulation check), the three facets reflecting the easiness effect (perceived study credibility, confidence in one’s ability to evaluate the study, and perceived ability to make decisions without further information, H1–H3), and intended behavioral reactions (knowledge-enhancing and social media reactions, H4, RQ1). The experiment ended with a debriefing.

### 2.5 Data analysis

First, a *t*-test for independent samples (with abstract video type as independent measure and perceived comprehensibility as dependent measure) was conducted to examine whether perceived comprehensibility was actually higher for PLS versus scientific abstracts (i.e., *manipulation check*). In case of significant differences between group variances (Levene’s test), adjusted degrees of freedom were used (Welch’s test). Since the sample size exceeded 30 persons per group, the *t*-test is robust to potential violations of the normal distribution assumption (Kubinger et al., 2009). Effect sizes were calculated using Hedge’s *g*, which is similar to Cohen’s *d* but takes into account different sample sizes between groups (as was the case in the present study). According to Cohen (1988), Hedge’s *g* or, alternatively, Cohen’s *d* around 0.2 indicate a small effect size, values around 0.5 a medium effect size, and values of at least 0.8 a large effect size. Additionally, an ANCOVA was performed to control for the potential effects of the two covariates (familiarity with psychological scientific studies and general ability to judge the veracity of psychological scientific studies).

Second, a 2 (video abstract type: PLS versus scientific abstracts) × 2 (debiasing video: shown versus not shown) between-participants ANOVA was calculated for each of the three facets reflecting the easiness effect, namely perceived study credibility (H1a, H2a, H3a), confidence in one’s ability to evaluate the studies (H1b, H2b, H3b), and perceived ability to make decisions without further information (H1c, H2c, H3c). All statistical requirements were sufficiently met, including no multicollinearity (*r* < 0.90, Verma, 2015), linearity (observed via scatterplots), and homogeneity of error variances (Levene’s tests, all *p*s > 0.10). The assumptions of normal distribution and extreme outliers were not tested as ANOVAs are robust to violations of the normal distribution assumption (Wilcox, 2011), with parametric tests being recommended when sample sizes of each group exceed *n* = 30 (Kubinger et al., 2009), and given the use of rating scales for the assessment of independent measures with extreme outliers not needing to be removed. Additionally, a 2 (abstract type) × 2 (debiasing video) ANCOVA with the two covariates (familiarity with psychological scientific studies, general ability to judge the veracity of psychological scientific studies) was calculated for each of the three facets of the easiness effect in order to explore whether this would change the results. Following the widely recognized recommendation of Simmons et al. (2011) to counteract increased false-positive results, we report the statistical results of the analyses with and without covariates. Effect sizes were calculated using partial eta squared, with η_p_^2^ around 0.01 reflecting small, η_p_^2^ around 0.06 medium, and η_p_^2^ greater than or equal to 0.14 reflecting large effect sizes (Cohen, 1988).

Third, *t*-tests for independent samples were computed for intended knowledge-enhancing reactions (H4a) and intended social media reactions (H4b), with video abstract type (PLS versus scientific abstracts) as independent variable. Statistical prerequisites were met, with non-significant Levene’s tests (both *p*s > 0.10) and sample sizes of each group exceeding *n* = 30 for the robustness of the normality assumption (Kubinger et al., 2009). Effect sizes were calculated using Hedge’s *g*.

Fourth, a *t*-test for independent samples was executed for intended knowledge-enhancing and social media reactions (RQ1), with debiasing video (shown versus not shown) as independent variable. Statistical requirements were tested and showed a non-significant Levene’s test (*p* > 0.10) and sample sizes greater than *n* = 30 (Kubinger et al., 2009).

All analyses were conducted using the statistical software program IBM SPSS Statistics 29.

## 3 Results

### 3.1 Manipulation check

To assess the manipulation check, a one-tailed *t*-test for independent samples was computed, using perceived comprehensibility as the dependent variable and video abstract type (PLS versus scientific abstracts) as the independent variable. On average, participants who received animated PLS (*M* = 5.68, *SD* = 0.79) rated the comprehensibility of such video abstracts higher compared to those who received animated scientific abstracts (*M* = 5.13, *SD* = 0.85), *t*(177) = 4.50, *p* < 0.001, *g* = 0.67, supporting a successful manipulation of the video abstracts’ comprehensibility. Importantly, comprehensibility was significantly different between video abstract types for all four videos (see Table 1). The ANCOVA comparing PLS with scientific abstracts and including the covariates—familiarity with psychological scientific studies (*M* = 3.49, *SD* = 1.53) and general ability to judge the veracity of psychological scientific studies (*M* = 3.90, *SD* = 1.30)—replicated the effect of video abstract type *F*(1, 175) = 20.60, *p* < 0.001, without significant effects of the covariates (familiarity: *p* = 0.330, ability to judge: *p* = 0.078).

**Table 1:** Means, standard deviations, and inferential statistics of the perceived comprehensibility of video abstracts (manipulation check).

Abstract comprehensibility	Video abstract type				*t*	*df*	*p*	Hedge’s *g*
	Animated PLS		Animated scientific abstracts					
	*M*	*SD*	*M*	*SD*				
All studies	5.68	0.79	5.13	0.85	4.50	177	<0.001	0.67
Kende et al. (2017)	5.43	1.15	4.40	1.41	5.41	176.99	<0.001	0.79
Bäck et al. (2018)	6.05	0.97	5.71	1.16	2.07	177	0.020	0.31
Halmburger et al. (2019)	5.41	1.27	5.05	1.30	1.85	177	0.033	0.28
Selvanathan and Lickel (2019)	5.84	1.02	5.35	1.34	2.79	175.82	0.003	0.41

### 3.2 H1: the easiness effect

We calculated a 2 (video abstract type: PLS versus scientific abstracts) × 2 (debiasing video: shown versus not shown) between-participants ANOVA. For a clear presentation, the corresponding main and interaction effects are presented separately according to hypotheses in the following three sections (3.2–3.4).

First, we examined the potential influence of the video abstract type on three facets reflecting the easiness effect: perceived study credibility (H1a), confidence in one’s ability to evaluate the study (H1b), and perceived ability to make decisions without further information (H1c). As summarized in Table 2, results showed that participants rated PLS, compared to scientific abstracts, significantly higher regarding perceived study credibility, *F*(1, 175) = 13.61, *p* < 0.001, η_p_^2^ = 0.072, and animated PLS also elicited a significantly higher confidence in the ability to evaluate the study, *F*(1, 175) = 4.99, *p* = 0.027, η_p_^2^ = 0.028. There was no significant difference between video abstract types in perceived ability to make decisions without further information, *F*(1, 175) = 1.73, *p* = 0.191, η_p_^2^ = 0.010. ANCOVAs including the covariates replicated these finding, with covariates being non-significant regarding all three facets (all *p*s > 0.05). In summary, the easiness effect was evident in animated video abstracts, because participants perceived higher study credibility (H1a) and confidence in their ability to evaluate the study (H1b) in case of animated PLS compared to animated scientific abstracts, but there was no significant difference in perceived ability to make decisions without further information (H1c).

**Table 2:** Effect of video abstract type (animated PLS versus animated scientific abstracts) on the three facets reflecting the easiness effect (first main effect of ANOVA).

Facet of the easiness effect	Video abstract type				*F*	*p*	η_p_^2^
	Animated PLS		Animated scientific abstracts				
	*M*	*SD*	*M*	*SD*			
Perceived study credibility (H1a)	5.16	1.08	4.56	1.01	13.61	<0.001	0.072
Confidence in one’s ability to evaluate the studies (H1b)	3.52	1.41	3.11	1.22	4.99	0.027	0.028
Perceived ability to make decisions without further information (H1c)	3.14	1.45	2.92	1.36	1.73	0.191	0.010

### 3.3 H2: the impact of a debiasing video on the easiness effect

H2 postulates that watching a debiasing video prior to receiving animated video abstracts reduces the easiness effect, that is, lower perceived study credibility (H2a), lower confidence in one’s ability to evaluate the study (H2b), and lower perceived ability to make decisions without further information (H2c). However, as shown in Table 3, we found no significant effects of the debiasing video on perceived study credibility, *F*(1, 175) = 1.07, *p* = 0.302, η_p_^2^ = 0.006, confidence in one’s ability to evaluate the study, *F*(1, 175) = 0.42, *p* = 0.837, η_p_^2^ < 0.001, and perceived ability to make decisions without further information, *F*(1, 175) = 0.001, *p* = 0.981, η_p_^2^ < 0.001. Again, ANCOVAs revealed no significant impact of covariates and replicated the results of the ANOVAs. In conclusion, the debiasing video did not have a significant effect on reducing the facets of the easiness effect.

**Table 3:** Effect of the debiasing video on the three facets reflecting the easiness effect (second main effect of ANOVA).

Facet of easiness effect	Debiasing video				*F*	*p*	η_p_^2^
	Shown		Not shown				
	*M*	*SD*	*M*	*SD*			
Perceived study credibility (H2a)	4.75	1.14	4.90	1.03	1.07	0.302	0.006
Confidence in one’s ability to evaluate the studies (H2b)	3.26	1.39	3.33	1.27	0.42	0.837	<0.001
Perceived ability to make decisions without further information (H2c)	2.99	1.41	3.03	1.40	0.001	0.981	<0.001

### 3.4 H3: interaction between abstract videos type and debiasing video

H3 postulated that perceived study credibility (H3a), confidence in one’s ability to evaluate the study (H3b), and perceived ability to make decisions without further information (H3c) are more strongly reduced by a debiasing video for participants who received animated PLS than for those who received animated scientific abstracts. However, the ANOVAs did not show significant interaction effects between the debiasing video (shown versus not shown) and the video abstract type (PLS versus scientific abstracts) on perceived study credibility, *F*(1, 175) = 0.57, *p* = 0.453, η_p_^2^ = 0.003, confidence in one’s ability to evaluate the study, *F*(1, 175) = 2.06, *p* = 0.153, η_p_^2^ = 0.012, and perceived ability to make decisions without further information, *F*(1, 175) = 3.40, *p* = 0.067, η_p_^2^ = 0.019. Again, the ANCOVAs replicated these non-significant effects. Therefore, the easiness effect was not more strongly reduced by a debiasing video for participants who received animated PLS than for participants who received animated scientific abstracts.

### 3.5 H4: impact of video abstract type on consumers reactions to video abstracts

H4a stated that participants differ in their amount of intended knowledge-enhancing reactions depending on the video abstract type they received. The average intention to carry out the specified reactions to animated PLS was *M* = 4.18 (*SD* = 1.31), and in case of animated scientific abstracts the average intention was *M* = 4.02 (*SD* = 1.22). The *t*-test showed that this difference was not significant, *t*(177) = 0.87, *p* = 0.388, *g* = 0.13. H4b stated that participants who received animated PLS and those who received animated scientific abstracts differ in terms of intended social media reactions to the video abstracts. The average intention to carry out the specified reactions to animated PLS was *M* = 2.78 (*SD* = 1.29) and to animated scientific abstracts was *M* = 2.64 (*SD* = 1.12). This difference was not significant, *t*(177) = 0.77, *p* = 0.441, *g* = 0.12. Importantly, the video abstract type showed no influence even at the level of individual reactions (see Supplementary Table B). In summary, the video abstract type had no influence on how strongly participants would react to the videos.

Regarding consumer reactions in general, the mean rating of intended knowledge-enhancing reactions to the video abstracts was *M* = 4.09 (*SD* = 1.26) and higher than the mean rating of intended social media reactions with *M* = 2.70 (*SD* = 1.20), suggesting a preference for knowledge-related forms of engagement—such as seeking additional information—over primarily communicative reactions like sharing or commenting. Moreover, knowledge-enhancing and social media reactions were positively correlated (*r* = 0.40, *p* < 0.001). For exploratory reasons, the complete intercorrelation matrix of the dependent variables can be found in Supplementary Table C.

### 3.6 RQ1: Impact of debiasing video on consumer reactions to video abstracts

Finally, the difference between participants who received a debiasing video prior to the video abstracts and participants who did not receive such a debiasing video were examined with respect to their intended consumer reactions to the video abstracts. Analyses showed no significant results: participants who received the debiasing video (*M* = 4.03, *SD* = 1.26) did not significantly differ in the extent of their intended knowledge-enhancing reactions from participants who did not receive the debiasing video (*M* = 4.14, *SD* = 1.27), *t*(177) = 0.55, *p* = 0.583, *g* = 0.083. Regarding intended social media reactions, participants who received the debiasing video (*M* = 2.71, *SD* = 1.26) did not significantly differ from participants who did not receive the debiasing video (*M* = 2.70, *SD* = 1.15), *t*(177) = −0.05, *p* = 0.961, *g* = −0.007. In sum, the debiasing video consistently showed no effects, not even on participants’ intended responses to the subsequently received video abstracts.

## 4 Discussion

The present study addressed a research gap by investigating the easiness effect in video abstracts, extending previous text-based research (e.g., Kerwer et al., 2021; Scharrer et al., 2013, 2014, 2017). We will discuss the results in detail in the following sections with their limitations and implications.

### 4.1 The easiness effect in video abstracts

The manipulation check showed that animated PLS were perceived as significantly more comprehensible than animated scientific abstracts. Although recent research demonstrated higher comprehensibility of written PLS compared to scientific abstracts (Kerwer et al., 2021; Stricker et al., 2020), its transfer to animations had so far remained an open question. The current finding underscores the potential of animated PLS as an effective tool for communicating scientific content to lay audiences and the broader public.

Moreover, to the best of our knowledge this study is the first to empirically demonstrate the easiness effect in video abstracts. Results revealed significant differences in two of the three facets reflecting the easiness effect, namely participants’ perceived study credibility and confidence in their ability to evaluate the presented study. This finding aligns with recent findings on the easiness effect in text-based research summaries (Kerwer et al., 2021; Scharrer et al., 2019; 2014, 2017).

Importantly, higher perceived credibility in the PLS condition supports the presence of the easiness effect—namely that content clarity and perceived comprehensibility can increase the credibility of scientific content. Simultaneously, this finding challenges the opposing scientificness effect, according to which more difficult scientific information leads to a higher credibility (Thomm and Bromme, 2011). In contrast to these results, Jonas et al. (2023) found that when directly comparing the influence of the scientificness effect (based on scientific features) with the easiness effect (based on linguistic accessibility and clarity), the scientificness effect had a significant impact on the perceived credibility of texts, whereas the easiness effect did not. More in line with our results, Jonas and Rosman (2024) showed that, in regard to enhancing credibility, the easiness effect can potentially offset the influence of the scienticficness effect, particularly in contexts of low scientific complexity. Hence, the occurrence of the easiness effect, but not the scientificness effect, in the present study could potentially be explained by our use of an animated format, which tends to reduce perceived complexity and scientific features due to its (cartoon) design. Overall, our findings suggest that—in context of animated formats—the aim to foster credibility within laypeople can be more effectively achieved through audio-visual simplification of information (as in the PLS) than through highlighting scientific features (as in the scientific abstracts). In this context, further examination of video abstracts with a focus on perceived scientificness would be a valuable approach for future research.

Higher confidence in one’s ability to evaluate scientific studies presented via PLS, compared to scientific abstracts, was also shown for text-based research summaries by research investigating the easiness effect (Kerwer et al., 2021; Scharrer et al., 2013, 2014). This heightened confidence highlights the empowering potential of PLS in general (Stoll et al., 2022), enabling lay audiences to feel more capable of engaging with scientific information. At the same time, it may also lead participants to overestimate their understanding and generalize trust in PLS, irrespective of study quality.

Contrary to our expectations, participants’ perceived ability to make decisions without further information was not significantly affected by the type of video abstract, and its mean value was the lowest of the three facets assumed to reflect the easiness effect. This suggests that participants were generally more cautious in this respect. A possible explanation for this finding is that this facet of the easiness effect implies a behavioral intention rather than a purely subjective evaluation of the presented scientific content. Making decisions involves a certain level of commitment, which participants might have been hesitant to express. Furthermore, the corresponding item includes the parenthesized information about consulting an expert (see Materials section), which may have primed participants to question their self-confidence when rejecting expert advice. Hence, the exact form of operationalization could have an influence on the sensitivity of the item to the manipulation at hand. In the present study, there was a descriptive difference in the assumed direction, but not strong enough to be statistically significant. In this context, it is worth mentioning that other operationalizations (cf. Mohajerzad et al., 2024; Scharrer et al., 2013, 2014, 2017) addressed individuals’ dependence on external expertise by specifying both the context (e.g., a specific claim) and the required judgment (e.g., evaluating its accuracy). In doing so, Mohajerzad et al. (2024) demonstrated the third facet of the easiness effect with significantly lower ratings for dependence on expertise when presented with an easy text compared to moderately and fully scientific texts.

Overall, the present results on the easiness effect support and extend existing literature. By examining video abstracts, the easiness effect could be shown for the first time in this new medium, which is becoming increasingly popular and attracting scientific attention (e.g., Bonnevie et al., 2023; Ferreira et al., 2021; Liu, 2022).

### 4.2 The robustness of the easiness effect: no effect of a debiasing intervention

Contrary to our assumption, the debiasing video did not reduce the easiness effect. This result is consistent with previous studies which also unsuccessfully attempted to eliminate the easiness effect by manipulating source credibility (Scharrer et al., 2019, 2022), controversial information presentation (Scharrer et al., 2014), or topic complexity (Scharrer et al., 2013).

Unlike these studies, the present study used a multimedia approach. Specifically, an animated debiasing video was developed, closely following successful debiasing strategies used to address other cognitive biases (Rusmana et al., 2020; Morewedge et al., 2015) and incorporating key principles of multimedia learning (Mayer, 2014). Despite these efforts, the video failed to achieve the desired reduction, although this intervention was of considerable length (4.5 min) and explained the easiness effect in great detail. Taken together, these findings underline the robustness of the easiness effect.

Nevertheless, some methodological aspects might have reduced the efficacy of the debiasing video. First, since the study was conducted remotely, participants might not have watched the debiasing video with sufficient attention. Second, the debiasing video might have been less effective due to its short duration compared to more extensive and intensive interventions including reflection exercises. Other studies (e.g., Morewedge et al., 2015; Dunbar et al., 2014; Rhodes et al., 2017; Rusmana et al., 2020) used longer videos and integrated structured elements such as everyday scenarios, reflection prompts, or follow-up tasks, whereas the debiasing video in the present study was relatively short, non-interactive, and did not include everyday context or prompts for reflection, which may also have limited its potential to engage system 2 thinking according to the two-system model of reasoning (Evans, 2003). In this respect, it is not possible to conclusively clarify whether the manipulation was merely too weak or whether a completely different form of intervention was needed. For example, approaches recommended by Scharrer et al. (2022)—such as educational interventions to help laypeople recognize the limitations of their understanding and to avoid relying solely on ease of comprehension as a validity cue or the combination of warning labels with critical thinking training—appear promising. However, there is still the possibility that the easiness effect is so robust that it cannot or can hardly be changed by intentional interventions.

### 4.3 Consumer reactions to video abstracts

Based on previous mixed findings, we explored whether receiving more comprehensible PLS, compared to scientific abstracts, would increase or reduce participants’ intention for consumer reactions to video abstracts. These reactions touch on the central function of scientific abstracts: stimulating further individual engagement with the research presented (knowledge-enhancing reactions) and/or interacting with the community about relevant content by social media reactions. We found that participants who received animated PLS and those who received animated scientific abstracts did not show significant differences in their intention to react to the video abstracts. This missing effect was consistently observed both at the level of the aggregated reactions and at the level of the individual reactions. These results therefore contradict the idea of a far-reaching easiness effect that also influences consumer reactions.

One possible explanation for these findings could be the interplay of competing mechanisms that may neutralize each other. For example, in terms of knowledge-enhancing reactions, PLS can be perceived as less scientific and less complex, leading participants to feel they do not require additional information; but at the same time, PLS may also be perceived as more engaging, potentially increasing interest and the striving for more content-related information. With respect to social media reactions, the lack of effect could be due to the fact that we only found a low overall intention to show corresponding reactions. These low intentions can likely be attributed to the widespread phenomenon of “lurkers”—online community members who do not actively participate in the communication process (Nonnecke and Preece, 1999). When baseline participation levels are low, significant differences between groups (animated PLS versus animated scientific abstracts) are therefore less likely to emerge.

Moreover, the aim of the debiasing video was to activate system 2 thinking, thereby raising awareness of cognitive biases and encouraging participants to seek additional information. Contrary to this, the debiasing video showed no effect on participants’ behavioral intentions to the video abstracts. Consequently, this intervention could neither influence the easiness effect nor modulate possible behavioral intentions. It can be assumed that the intervention was either too weak to activate system 2 thinking, so that the assumed process was not triggered. Alternatively, other mediating variables that were not taken into account could also play a role in whether an informative video (debiasing video) before the reception of the target content (video abstracts) influences the subsequent handling of this content in the sense of further knowledge gathering (knowledge-enhancing reactions) or rather of interpersonal communicative behavior (social media reactions) at all.

Finally, while there were no differences in knowledge-enhancing reactions and social media reactions between participants who received PLS compared to scientific abstracts, notable distinctions emerged in the patterns of correlations (see Supplementary Table C). Knowledge-enhancing reactions and social media reactions were positively correlated with the comprehensibility of the video abstracts. This finding is consistent with the fluency processing assumption (Reber et al., 1998) and motivational aspects of cognitive load theory (Evans et al., 2024): When content is easier to understand, it creates a sense of ease and enjoyment, fostering a positive experience and increasing motivation to engage further. In contrast, only social media reactions were positively correlated with participants’ confidence in their ability to evaluate the study and the perceived ability to make decisions without further information. This connection seems understandable as social media reactions are visible to other people and therefore also have a strong communicative function. The stronger one’s own feeling of being able to evaluate the study presented in the video abstract and making a decision based on the reported content, the more inclined people obviously are to evaluate, comment on, and share this content with their community. Nonetheless, to better understand these inter-correlations, future research should focus on additional variables. Previous studies in the field of (multimedia) learning (e.g., Ismail et al., 2013; Moreno and Mayer, 2007) have shown that variables such as interest, students’ beliefs, and motivation significantly affected engagement and effort investment. Potential other factors may include personal preferences (e. g., interest in the topic, topic relevance, and thematic appeal) and metacognitive elements (e.g., cognitive load, need for cognition), alongside other potentially moderating variables (e. g., quality of content, social media usage habits, social norms) that could shape the relationship between content characteristics and behavioral intentions. Future research should integrate these variables to provide a more comprehensive understanding of behavioral reactions in science communication.

### 4.4 Limitations and future research

The present study has some limitations that should be acknowledged.

First, the research setting was not fully controlled, as participants completed the study in an unsupervised setting. This may have influenced their attention and engagement with the videos. Although participants with significantly prolonged processing times were excluded, uninterrupted attention could not be ensured for all participants. Future studies in a laboratory setting could provide greater control over participant behavior and engagement. However, we decided to realize this study as an online experiment in order to achieve high ecological validity of the results. People usually consume content on their own (mobile) devices in an environment of their choice. We therefore asked whether the easiness effect can occur in this context, which the results confirm, and whether an intervention (debiasing video) could have an effect in this context, which we did not find.

Second, nevertheless, the study’s ecological validity was still limited, particularly regarding participants’ reactions to the videos. The use of self-reported behavioral intentions without a specific context (e.g., platform information) may not fully capture real-life responses. Future studies could address this by designing experiments based on real-world platforms, such as YouTube, to better assess participants’ actual reactions. However, a fundamental problem is that social media reactions hardly work in an artificially constructed virtual space. For this to work, it would have to be possible to reach the participants’ real social network via the responses offered in order to reflect their individual communicative reality. With respect to knowledge-enhancing reactions, an implementation seems less demanding. For example, offering participants a download link for the full text after receiving a (video) abstract could operationalize the whish for full-text access. Also, offering additional videos can be easily implemented, but may require a much greater amount of resources to produce the corresponding (video) content.

Third, the sample in this study lacked diversity in educational background, consisting predominantly of participants with a strong academic background. This limits the generalizability of the findings. We specifically acquired such a sample because their fundamental interest in scientific information formed an important basis for the present study. And, as Andreassen et al. (2007) reported, young educated individuals search for scientific information online most frequently. In fact, as noted by Stoll et al. (2022), studies on science communication using PLS often involve small, highly educated samples. Future research should aim to include larger and more diverse samples, to enhance the generalizability of findings to broader populations.

Fourth, previous research, including the present study, demonstrated the easiness effect while primarily focusing on experimentally manipulating the content characteristics of the stimulus material, whereas the influence of potential moderator variables was not explicitly investigated. Particularly, as belief consistency was shown to have a significant influence on the easiness effect (Scharrer et al., 2021), prior beliefs could be included as a promising moderator variable in future research. Another potential moderator variable in context of the credibility of science-related content could be political orientation, which was shown to be strongly correlated with trust in scientists (Cologna et al., 2025). Future research should also compare the easiness effect across different modalities (i.e., video versus text-based) in controlled (quasi-)experimental designs. In the field of education research, comparisons between video-based and text-based learning material for knowledge transfer has gained significant attention in the last years (cf. Kramer et al., 2020). Given the resource-intensive nature of producing animated videos (e.g., time, software, hardware, creativity), studies should evaluate the advantages and limitations of this format relative to text-based communication. Generative artificial intelligence (AI) offers a promising, resource-efficient approach to enhancing science communication. AI-generated texts are more readable, accessible, and effective in conveying scientific ideas, leading to better comprehension and more accurate summaries, but also contribute to the easiness effect (Markowitz, 2024). In the context of AI, ethical concerns like transparency, representation bias, and potential loss of nuance highlight the need for robust evaluation strategies and quality control in future research.

### 4.5 Conclusion

The present study addressed a research gap by investigating the easiness effect in video abstracts, extending previous text-based research. The use of such a multimedia approach for disseminating scientific content is in line with findings and suggestions of Apaydin et al. (2024), who identified best practices for PLS, emphasizing the integration of media formats like infographics, audio tracks, and short interactive videos to enhance engagement, communication effectiveness, and participant involvement. The present study offers valuable insights into animated videos, highlighting the persistence of the easiness effect, the ineffectiveness of a one-shot video-based debiasing intervention, and the absence of significant differences in participants’ intended reactions to video abstract types (PLS versus scientific abstracts).

As animated videos and especially video abstracts are becoming increasingly popular as a means of presenting scientific content and addressing complex topics, it is essential to explore their opportunities and challenges. On the one hand, simplified research summaries may lead to overconfidence in the ability to evaluate scientific findings, potentially contributing to the spread of misinformation and fake news. On the other hand, videos and social media platforms offer great potential for promoting science communication and engaging the broader public. Further research is needed to identify factors that influence consumer reactions to scientific content. The scientific community must rise to the challenge of effectively educating the public on critical findings while reducing the tendency to overestimate their own competence.

## Data Availability

The raw data supporting the conclusions of this article will be made available by the authors, without undue reservation.

## References

[ref1] Andreassen H. K. Bujnowska-Fedak M. M. Chronaki C. E. Dumitru R. C. Pudule I. Santana S.. (2007). European citizens’ use of e-health services: a study of seven countries. BMC Public Health 7:53. doi: 10.1186/1471-2458-7-53, PMID: 17425798 PMC1855923

[ref2] Apaydin N. Crosthwait R. Pire-Smerkanich N. (2024). Identifying best practices: content analysis of plain language summary (PLS) resources for disseminating study results to participants. J. Clin. Transl. Sci. 8, 160–161. doi: 10.1017/cts.2024.460

[ref3] Audacity Team. (2021). Audacity (version 3.0.0) [computer software]. Available online at: https://www.audacityteam.org/.

[ref4] Bäck E. A. Bäck H. Sendén M. G. Sikström S. (2018). From I to we: group formation and linguistic adaption in an online xenophobic forum. J. Soc. Polit. Psychol. 6, 76–91. doi: 10.5964/jspp.v6i1.741

[ref5] Barnes A. Patrick S. (2019). Lay summaries of clinical study results: an overview. Pharmaceut. Med. 33, 261–268. doi: 10.1007/s40290-019-00285-0, PMID: 31933186

[ref6] Bonnevie T. Repel A. Gravier F. E. Ladner J. Sibert L. Muir J. F.. (2023). Video abstracts are associated with an increase in research reports citations, views and social attention: a cross-sectional study. Scientometrics 128, 3001–3015. doi: 10.1007/s11192-023-04675-9, PMID: 37101977 PMC10028770

[ref7] Bredbenner K. Simon S. M. (2019). Video abstracts and plain language summaries are more effective than graphical abstracts and published abstracts. PLoS One 14:e0224697. doi: 10.1371/journal.pone.0224697, PMID: 31743342 PMC6863540

[ref8] Bromme R. Scharrer L. Stadtler M. Hömberg J. Torspecken R. (2015). Is it believable when it’s scientific? How scientific discourse style influences laypeople’s resolution of conflicts. J. Res. Sci. Teach. 52, 36–57. doi: 10.1002/tea.21172

[ref9] Buljan I. Malički M. Wager E. Puljak L. Hren D. Kellie F.. (2018). No difference in knowledge obtained from infographic or plain language summary of a Cochrane systematic review: three randomized controlled trials. J. Clin. Epidemiol. 97, 86–94. doi: 10.1016/j.jclinepi.2017.12.003, PMID: 29269021

[ref10] Bullock O. M. Colón Amill D. Shulman H. C. Dixon G. N. (2019). Jargon as a barrier to effective science communication: evidence from metacognition. Public Underst. Sci. 28, 845–853. doi: 10.1177/0963662519865687, PMID: 31354058

[ref11] Cinelli M. Quattrociocchi W. Galeazzi A. Valensise C. M. Brugnoli E. Schmidt A. L.. (2020). The COVID-19 social media infodemic. Sci. Rep. 10, Article 16598:16598. doi: 10.1038/s41598-020-73510-5, PMID: 33024152 PMC7538912

[ref12] Cohen J. (1988). Statistical power analysis for the behavioral sciences. 2nd Edn. Hillsdale, NJ: Lawrence Erlbaum Associates.

[ref13] Cologna V. Mede N. G. Berger S. Besley J. Brick C. Joubert M.. (2025). Trust in scientists and their role in society across 68 countries. Nat. Hum. Behav. 9, 713–730. doi: 10.1038/s41562-024-02090-5, PMID: 39833424 PMC7617525

[ref14] Dunbar N. E. Miller C. H. Adame B. J. Elizondo J. Wilson S. N. Lane B. L.. (2014). Implicit and explicit training in the mitigation of cognitive bias through the use of a serious game. Comput. Hum. Behav. 37, 307–318. doi: 10.1016/j.chb.2014.04.053

[ref15] Evans J. S. B. (2003). In two minds: dual-process accounts of reasoning. Trends Cogn. Sci. 7, 454–459. doi: 10.1016/j.tics.2003.08.012, PMID: 14550493

[ref16] Evans P. Vansteenkiste M. Parker P. Kingsford-Smith A. Zhou S. (2024). Cognitive load theory and its relationships with motivation: a self-determination theory perspective. Educ. Psychol. Rev. 36:7. doi: 10.1007/s10648-023-09841-2

[ref17] Eysenbach G. (2020). How to fight an infodemic: the four pillars of infodemic management. J. Med. Internet Res. 22:e21820. doi: 10.2196/21820, PMID: 32589589 PMC7332253

[ref18] Falk J. Dierking L. Schwanger L. P. Status N. Back M. Barriault C.. (2016). Correlating science center use with adult science literacy: an international, cross-institutional study. Sci. Educ. 100, 849–876. doi: 10.1002/sce.21225

[ref19] Farinella M. (2018). The potential of comics in science communication. J. Sci. Commun. 17:Y01. doi: 10.22323/2.17010401

[ref20] Faul F. Erdfelder E. Buchner A. Lang A.-G. (2009). Statistical power analyses using G* power 3.1: tests for correlation and regression analyses. Behav. Res. Methods 41, 1149–1160. doi: 10.3758/BRM.41.4.1149, PMID: 19897823

[ref21] Ferreira M. Lopes B. Granado A. Freitas H. Loureiro J. (2021). Audio-visual tools in science communication: the video abstract in ecology and environmental sciences. Front. Commun. 6:596248. doi: 10.3389/fcomm.2021.596248

[ref22] Fitz Gibbon H. King K. Piano C. Wilk C. Gaskarth M. (2020). Where are biomedical research plain-language summaries? Health. Sci. Rep. 3:e175. doi: 10.1002/hsr2.175, PMID: 32789193 PMC7416593

[ref23] Fraser N. Brierley L. Dey G. Polka J. K. Pálfy M. Nanni F.. (2021). The evolving role of preprints in the dissemination of COVID-19 research and their impact on the science communication landscape. PLoS Biol. 19:e3000959. doi: 10.1371/journal.pbio.3000959, PMID: 33798194 PMC8046348

[ref24] Glaser M. Schwan S. (2015). Explaining pictures: how verbal cues influence processing of pictorial learning material. J. Educ. Psychol. 107, 1006–1018. doi: 10.1037/edu0000044

[ref25] GoAnimate, Inc. (2022). VYOND studio [online software]. Available at: https://vyond.com.

[ref26] Goldman S. R. Bisanz G. L. (2002). “Toward a functional analysis of scientific genres: implications for understanding and learning processes” in The psychology of science text comprehension. eds. Otero J. León J. A. Graesser A. C. (Mahwah, NJ: Lawrence Erlbaum Associates Publishers), 19–50.

[ref27] Halmburger A. Baumert A. Rothmund T. (2019). Seen one, seen em all? Do reports about law violations of a single politician impair the perceived trustworthiness of politicians in general and of the political system? J. Soc. Polit. Psychol. 7, 448–477. doi: 10.5964/jspp.v7i1.933

[ref28] Hansen J. Dechêne A. Wänke M. (2008). Discrepant fluency increases subjective truth. J. Exp. Soc. Psychol. 44, 687–691. doi: 10.1016/j.jesp.2007.04.005

[ref29] Hargittai E. Füchslin T. Schäfer M. S. (2018). How do young adults engage with science and research on social media? Some preliminary findings and an agenda for future research. Soc. Media Soc. 4, 1–10. doi: 10.1177/2056305118797720, PMID: 40441278

[ref30] Hauck S. A. (2019). Sharing planetary science in plain language. J. Geophys. Res. Planets 124, 2462–2464. doi: 10.1029/2019JE006152

[ref31] Ismail H. N. Kuldas S. Hamzah A. (2013). Do students need more motivational resources or more cognitive resources for better learning? Procedia Soc. Behav. Sci. 97, 325–332. doi: 10.1016/j.sbspro.2013.10.241

[ref32] Jonas M. Kerwer M. Chasiotis A. Rosman T. (2023). Indicators of trustworthiness in lay-friendly research summaries: Scientificness surpasses easiness. Public Underst. Sci. 33, 37–57. doi: 10.1177/09636625231176377, PMID: 37278009 PMC10756015

[ref33] Jonas M. Rosman T. (2024). All research summaries are scientific, but some are more scientific than others? Indications for a mediation of the relationship between author and text scientificness through perceived scientificness. Collabra: Psychology 10:123196. doi: 10.1525/collabra.123196, PMID: 34375835

[ref34] Joubert M. Davis L. Metcalfe J. (2019). Storytelling: the soul of science communication. J. Sci. Commun. 18:Article E. doi: 10.22323/2.18050501

[ref35] Kahneman D. (2003). A perspective on judgment and choice: mapping bounded rationality. Am. Psychol. 58, 697–720. doi: 10.1037/0003-066X.58.9.697, PMID: 14584987

[ref36] Kaspar K. Wehlitz T. von Knobelsdorff S. Wulf T. von Saldern M. A. O. (2015). A matter of font type: the effect of serifs on the evaluation of scientific abstracts. Int. J. Psychol. 50, 372–378. doi: 10.1002/ijop.12160, PMID: 25704872

[ref37] Katz E. Blumler J. G. Gurevitch M. (1973). Uses and gratifications research. Public Opin. Q. 37, 509–523. doi: 10.1086/268109

[ref38] Kende A. Lantos N. A. Belinszky A. Csaba S. Lukács Z. A. (2017). The politicized motivations of volunteers in the refugee crisis: intergroup helping as the means to achieve social change. J. Soc. Polit. Psychol. 5, 260–281. doi: 10.5964/jspp.v5i1.642

[ref39] Kerwer M. Chasiotis A. Stricker J. Günther A. Rosman T. (2021). Straight from the scientist’s mouth–plain language summaries promote laypeople’s comprehension and knowledge acquisition when reading about individual research findings in psychology. Collabra: Psychology 7:Article 18898. doi: 10.1525/collabra.18898, PMID: 34375835

[ref40] Khan M. L. (2017). Social media engagement: what motivates user participation and consumption on YouTube? Comput. Hum. Behav. 66, 236–247. doi: 10.1016/j.chb.2016.09.024

[ref41] Kramer C. König J. Strauss S. Kaspar K. (2020). Classroom videos or transcripts? A quasi-experimental study to assess the effects of media-based learning on pre-service teachers’ situation-specific skills of classroom management. Int. J. Educ. Res. 103:101624. doi: 10.1016/j.ijer.2020.101624

[ref42] Kubinger K. D. Rasch D. Moder K. (2009). Zur Legende der Voraussetzungen des t-tests für unabhängige Stichproben [legend for the prerequisites of the t-test for independent samples]. Psychol. Rundsch. 60, 26–27. doi: 10.1026/0033-3042.60.1.26

[ref43] Kuehne L. M. Olden J. D. (2015). Lay summaries needed to enhance science communication. Proc. Natl. Acad. Sci. USA 112, 3585–3586. doi: 10.1073/pnas.1500882112, PMID: 25805804 PMC4378425

[ref44] Laine C. Goodman S. N. Griswold M. E. Sox H. C. (2007). Reproducible research: moving toward research the public can really trust. Ann. Intern. Med. 146, 450–453. doi: 10.7326/0003-4819-146-6-200703200-00154, PMID: 17339612

[ref45] Levie W. H. Lentz R. (1982). Effects of text illustrations: a review of research. Educ. Technol. Res. Dev. 30, 195–232. doi: 10.1007/BF02765184, PMID: 40448853

[ref46] Liu J. (2022). Video or perish? An analysis of video abstract author guidelines. J. Librariansh. Inf. Sci. 54, 230–238. doi: 10.1177/09610006211006774

[ref47] Markowitz D. M. (2024). From complexity to clarity: how AI enhances perceptions of scientists and the public’s understanding of science. PNAS Nexus 3:387. doi: 10.1093/pnasnexus/pgae387, PMID: 39290437 PMC11406778

[ref48] Mayer R. E. (1989). Systematic thinking fostered by illustrations in scientific text. J. Educ. Psychol. 81, 240–246. doi: 10.1037/0022-0663.81.2.240

[ref49] Mayer R. E. (2001). Multimedia learning. Cambridge, UK: Cambridge University Press.

[ref50] Mayer R. E. (Ed.). (2005). Cambridge handbook of multimedia learning. Cambridge University Press. Available online at: https://psycnet.apa.org/record/2006-00633-000.

[ref51] Mayer R. E. (2014). The Cambridge handbook of multimedia learning. Cambridge, UK: Cambridge University Press.

[ref52] Mayer R. E. Anderson R. B. (1992). The instructive animation: helping students build connections between words and pictures in multimedia learning. J. Educ. Psychol. 84, 444–452. doi: 10.1037/0022-0663.84.4.444

[ref53] Mayer R. E. Gallini J. K. (1990). When is an illustration worth ten thousand words? J. Educ. Psychol. 82, 715–726. doi: 10.1037/0022-0663.82.4.715

[ref54] Mayer R. E. Moreno R. (2002). Animation as an aid to multimedia learning. Educ. Psychol. Rev. 14, 87–99. doi: 10.1023/A:1013184611077

[ref55] McQuail D. (Ed.). (1987). Mass communication theory: An introduction 2. Sage Publications. Available online at: https://psycnet.apa.org/record/1987-98365-000.

[ref56] Milkman K. L. Chugh D. Bazerman M. H. (2009). How can decision making be improved? Perspect. Psychol. Sci. 4, 379–383. doi: 10.1111/j.1745-6924.2009.01142.x 26158985

[ref57] Mohajerzad H. Martin A. Kamphausen L. Widany S. (2024). Communicating research to practitioners – between scientific rigor, easy science and practitioners’ self-perception of expertise. J. Prof. Capital Commun. 9, 196–210. doi: 10.1108/JPCC-01-2024-0003

[ref58] Möller A. M. Kühne R. Baumgartner S. E. Peter J. (2019). Exploring user responses to entertainment and political videos: an automated content analysis of YouTube. Soc. Sci. Comput. Rev. 37, 510–528. doi: 10.1177/0894439318779336

[ref59] Moreno R. Mayer R. (2007). Interactive multimodal learning environments. Educ. Psychol. Rev. 19, 309–326. doi: 10.1007/s10648-007-9047-2

[ref60] Morewedge C. K. Kahneman D. (2010). Associative processes in intuitive judgment. Trends Cogn. Sci. 14, 435–440. doi: 10.1016/j.tics.2010.07.004, PMID: 20696611 PMC5378157

[ref61] Morewedge C. K. Yoon H. Scopelliti I. Symborski C. W. Korris J. H. Kassam K. S. (2015). Debiasing decisions: improved decision making with a single training intervention. Policy Insights Behav. Brain Sci. 2, 129–140. doi: 10.1177/2372732215600886

[ref62] Nonnecke B. Preece J. (1999). Shedding light on lurkers in online communities. Ethnographic studies in real and virtual environments: Inhabited information spaces and connected communities, Edinburgh. Available online at: https://www.semanticscholar.org/paper/Shedding-Light-on-Lurkers-in-Online-CommunitiesNonnecke-Preece/e7dde9c3daebb20ae54b599a2ac95350d80397f4#paper-header.

[ref63] Osman W. Mohamed F. Elhassan M. Shoufan A. (2022). Is YouTube a reliable source of health-related information? A systematic review. BMC Med. Educ. 22:382. doi: 10.1186/s12909-022-03446-z, PMID: 35590410 PMC9117585

[ref64] Pitcher N. Mitchell D. Hughes C. (2022). Template and guidance for writing a Cochrane plain language summary. Cochrane Collaboration. Available online at: https://training.cochrane.org/guidance-writing-cochrane-plain-language-summary.

[ref65] Reber R. Schwarz N. (1999). Effects of perceptual fluency on judgments of truth. Conscious. Cogn. 8, 338–342. doi: 10.1006/ccog.1999.0386, PMID: 10487787

[ref66] Reber R. Winkielman P. Schwarz N. (1998). Effects of perceptual fluency on affective judgments. Psychol. Sci. 9, 45–48. doi: 10.1111/1467-9280.00008

[ref67] Rhodes R. E. Kopecky J. Bos N. McKneely J. Gertner A. Zaromb F.. (2017). Teaching decision making with serious games: an independent evaluation. Games Cult. 12, 233–251. doi: 10.1177/1555412016686642

[ref68] Rosenthal S. (2018). Motivations to seek science videos on YouTube: free-choice learning in a connected society. Int. J. Sci. Educ. 8, 22–39. doi: 10.1080/21548455.2017.1371357, PMID: 40406382

[ref69] Rusmana A. N. Roshayanti F. Ha M. (2020). Debiasing overconfidence among indonesian undergraduate students in the biology classroom: an intervention study of the KAAR model. Asia Pac. Sci. Educ. 6, 228–254. doi: 10.1163/23641177-BJA00001

[ref70] Salzmann S. (2025). Easiness effect in animated research summaries. OSF. Available online at: https://osf.io/386bq/?view_only=440ad7129310402ab90d5bc213f9a4f6 (Retrieved February 7, 2025).

[ref71] Santesso N. Rader T. Nilsen E. S. Glenton C. Rosenbaum S. Ciapponi A.. (2015). A summary to communicate evidence from systematic reviews to the public improved understanding and accessibility of information: a randomized controlled trial. J. Clin. Epidemiol. 68, 182–190. doi: 10.1016/j.jclinepi.2014.04.009, PMID: 25034199

[ref72] Scharrer L. Britt M. A. Stadtler M. Bromme R. (2013). Easy to understand but difficult to decide: information comprehensibility and controversiality affect laypeople’s science-based decisions. Discourse Process. 50, 361–387. doi: 10.1080/0163853X.2013.813835

[ref73] Scharrer L. Bromme R. Britt M. A. Stadtler M. (2012). The seduction of easiness: how science depictions influence laypeople’s reliance on their own evaluation of scientific information. Learn. Instr. 22, 231–243. doi: 10.1016/j.learninstruc.2011.11.004

[ref74] Scharrer L. Bromme R. Stadtler M. (2021). Information easiness affects non-experts’ evaluation of scientific claims about which they hold prior beliefs. Front. Psychol. 12:678313. doi: 10.3389/fpsyg.2021.678313, PMID: 34512439 PMC8430255

[ref75] Scharrer L. Pape V. Stadtler M. (2022). Watch out: fake! How warning labels affect laypeople’s evaluation of simplified scientific misinformation. Discourse Process. 59, 575–590. doi: 10.1080/0163853X.2022.2096364

[ref76] Scharrer L. Rupieper Y. Stadtler M. Bromme R. (2017). When science becomes too easy: science popularization inclines laypeople to underrate their dependence on experts. Public Underst. Sci. 26, 1003–1018. doi: 10.1177/0963662516680311, PMID: 27899471

[ref77] Scharrer L. Stadtler M. Bromme R. (2014). You’d better ask an expert: mitigating the comprehensibility effect on laypeople’s decisions about science-based knowledge claims. Appl. Cogn. Psychol. 28, 465–471. doi: 10.1002/acp.3018

[ref78] Scharrer L. Stadtler M. Bromme R. (2019). Judging scientific information: does source evaluation prevent the seductive effect of text easiness? Learn. Instr. 63:101215. doi: 10.1016/j.learninstruc.2019.101215

[ref79] Selvanathan H. P. Lickel B. (2019). A field study around a racial justice protest on a college campus: the proximal impact of collective action on the social change attitudes of uninvolved bystanders. J. Soc. Polit. Psychol. 7, 598–619. doi: 10.5964/jspp.v7i1.1063

[ref80] Shao G. (2009). Understanding the appeal of user-generated media: a uses and gratification perspective. Internet Res. 19, 7–25. doi: 10.1108/10662240910927795

[ref81] Shoufan A. (2019). Estimating the cognitive value of YouTube’s educational videos: a learning analytics approach. Comput. Hum. Behav. 92, 450–458. doi: 10.1016/j.chb.2018.03.036

[ref82] Shoufan A. Mohamed F. (2022). YouTube and education: a scoping review. IEEE Access 10, 125576–125599. doi: 10.1109/ACCESS.2022.3225419

[ref83] Simmons J. P. Nelson L. D. Simonsohn U. (2011). False-positive psychology: undisclosed flexibility in data collection and analysis allows presenting anything as significant. Psychol. Sci. 22, 1359–1366. doi: 10.1177/0956797611417632, PMID: 22006061

[ref84] Sorden S. D. (2012). “A cognitive theory of multimedia learning” in The handbook of educational theory. eds. Irby B. J. Brown G. Lara-Alecio R. Jackson S.. (Charlotte, NC: Information Age Publishing), 41–62.

[ref85] Stoll M. Kerwer M. Lieb K. Chasiotis A. (2022). Plain language summaries: a systematic review of theory, guidelines and empirical research. PLoS One 17:e0268789. doi: 10.1371/journal.pone.0268789, PMID: 35666746 PMC9170105

[ref86] Stricker J. Chasiotis A. Kerwer M. Günther A. (2020). Scientific abstracts and plain language summaries in psychology: a comparison based on readability indices. Plo S One 15:e0231160. doi: 10.1371/journal.pone.0231160, PMID: 32240246 PMC7117690

[ref87] SurveyCircle. (2016). SurveyCircle [website]. Mannheim. Available online at: https://www.surveycircle.com.

[ref88] Takahashi B. Tandoc E. C. Jr. (2016). Media sources, credibility, and perceptions of science: learning about how people learn about science. Public Underst. Sci. 25, 674–690. doi: 10.1177/0963662515574986, PMID: 25792288

[ref89] Thomm E. Bromme R. (2011). “It should at least seem scientific!” textual features of “scientificness” and their impact on lay assessments of online information. Sci. Educ. 96, 187–211. doi: 10.1002/sce.20480, PMID: 40450705

[ref90] Thon F. M. Jucks R. (2017). Believing in expertise: how authors’ credentials and language use influence the credibility of online health information. Health Commun. 32, 828–836. doi: 10.1080/10410236.2016.1172296, PMID: 27466693

[ref91] Tivian XI GmbH. (2022). Unipark. (Version 7.0) [Computer software]. EFS Survey Software.

[ref92] Verma J. P. (2015). Repeated measures design for empirical researchers. 1. Wiley. Available online at: https://www.wiley-vch.de/de/fachgebiete/mathematik-und-statistik/repeated-measures-designfor-empirical-researchers-978-1-119-05271-5.

[ref93] Wilcox R. R. (2011). Introduction to robust estimation and hypothesis testing. San Diego, CA: Academic Press.

[ref94] Winkielman P. Cacioppo J. T. (2001). Mind at ease puts a smile on the face: psychophysiological evidence that processing facilitation elicits positive affect. J. Pers. Soc. Psychol. 81, 989–1000. doi: 10.1037/0022-3514.81.6.989, PMID: 11761320

[ref95] Zimmermann D. Noll C. Gräßer L. Hugger K. U. Braun L. M. Nowak T.. (2020). Influencers on YouTube: a quantitative study on young people’s use and perception of videos about political and societal topics. Curr. Psychol. 41, 6808–6824. doi: 10.1007/s12144-020-01164-7, PMID: 40448853

