# Supplementary material for: A new dimension of simplified science communication: the easiness effect of science popularization in animated video abstracts
Source: Front Psychol. 2025 Jul 2;16:1584695. doi: 10.3389/fpsyg.2025.1584695 (PMC12263670; doi:10.3389/fpsyg.2025.1584695)
Supplement: Supplementary file 1 [file Data_Sheet_1.pdf]

Supplementary Material

A new dimension of simplified science communication: The easiness effect of science popularization in animated video abstracts

Sara Salzmann, Charlotte Walther, Kai Kaspar

1 Supplementary Material A

Scientific abstracts and plain language summaries

The abstracts and PLS on which the animated videos of the present study were based were published in the Journal of Social and Political Psychology (https://jspp.psychopen.eu/index.php/jspp). The abstracts and PLS are freely available under a CC-BY license (https://creativecommons.org/licenses/by/4.0/). Below, the original abstracts and PLS of the four studies used in the present study are listed. Subheadings/intermediate questions of the original PLS were not included to keep the one speaker role in the associated video abstracts consistent for scientific abstracts and PLS.

1.1 Study A

Scientific abstract

"By bringing together a sophisticated conceptualization of political trustworthiness (integrated model of trust) with theorizing from information processing (trait inferences, inclusion-exclusion model), our research aimed at investigating the impact of a politician's unlawful behavior on political trust. In four experimental studies, we investigated how laypersons draw inferences from media reports about a politician's law violation to the trustworthiness of (a) that politician, (b) politicians in general, and (c) the political system as a whole. Participants who read a bogus newspaper report about a violation of law (child pornography or financial fraud) ascribed lower integrity, benevolence, and competence to the respective politician compared to those in a control condition (Study 1, 3, & 4). The perceived trustworthiness of politicians in general and the political system was also found to be decreased in one study (Study 2), which did not include items asking for the trustworthiness of the law-violating politician. By contrast, two studies including such items revealed only indirect effects through the perceived trustworthiness of the politician in question (Study 3 & 4). Our results suggest that law violations negatively affect the responsible politicians. In line with the inclusion-exclusion model, the impact from the wrongdoing of one politician to all politicians or the political system seems to be highly influenced by boundary conditions." (Halmburger et al., 2019, p. 448)

Correspondending Plain Language Summary

"Although trust in politicians seems to decline across several countries in Europe and in the United States, the reasons for these trends have not yet sufficiently been investigated. As negative media report about politicians have repeatedly been discusses as causes for these trends, we investigated their effects on political trustworthiness in Germany.

Political trust builds one cornerstone of democratic systems. It empowers a functional political system through the legitimization of its representatives as well as political engagement and voting behavior of its citizens. Thereby, political trust embraces how much citizens trust the political system in general, political institutions, politicians in general as well as specific politicians. As some of the subordinate agents of trustworthiness represent the superior categories, they cannot be seen as fully independent concepts. By investigating the impact of the illegal behavior of a specific politician on politicians in general and the political system, we can enhance our understanding of the interconnectedness of these different levels of political trust, but also if media reports can be seen as one cause for a general decline of political trust. Previous research primarily investigated real, but isolated cases of political scandals that happened in the past. Our research complements their findings by focusing on immediate reactions towards new cases. Importantly, we varied the type of illegal behavior and office of the involved politician to extend the generalizability of our results.

Four empirical studies with together 950 participants have been conducted in Germany. All participants either read a fictitious article about a politician's illegal or legal behavior. We assessed their perceived integrity, benevolence, and competence of the respective politician. By this, we aimed at exploring out how much citizens' generalize from the illegal behavior to all three dimensions of perceived trustworthiness of the politician. In addition, we investigated whether reading about a single politician's wrongdoing affects the perceived trustworthiness of politicians in general and the political system. Whereas our results clearly supported that illegal behavior of a politician decreases how integer, benevolent, and competent he is perceived, results regarding politicians in general and the system were mixed.

Our research supported the idea that media reports about a politician's illegal behavior strongly damage this politician's perception. With regard to all politicians and the political system, the kind of impact seems to be much more dependent of specific boundary conditions. It seems that several processes can buffer the negative effect of the decreased trustworthiness of a single politician. Although we can only speculate on these processes at the moment, our research provides the foundation for previous research that aims at revealing the interconnectedness and dynamics of different levels of political trust." (Halmburger et al., 2019, p. 448)

Reference

Halmburger, A., Baumert, A., & Rothmund, T. (2019). Seen one, seen 'em all? Do reports about law violations of a single politician impair the perceived trustworthiness of politicians in general and of the political system?. *Journal of Social and Political Psychology*, *7*(1), 448–477. https://doi.org/10.5964/jspp.v7i1.933

1.2 Study B

Scientific abstract

"Social movements often use protests and other collective actions to draw public attention to their cause, yet the psychological reactions to such actions from their targeted audience is not well understood. This research investigates uninvolved bystanders' immediate responses to collective action using a quasi-experimental field study designed around a racial justice protest that took place at a large public university in the United States. We surveyed two student samples exactly one week apart at the same time and location, first in the absence of protest and then again at the time of a racial justice protest (Total N = 240). We found that participants who believed that racism was not a problem on campus had more negative attitudes toward racial justice protests and protesters, as well as lower support for anti-racist efforts on campus on the day of the protest, compared to the day without a protest. These findings provide initial evidence that a protest encounter may trigger a backlash effect amongst those who have the most resistant attitudes toward social change." (Selvanathan & Lickel, 2019, p. 598)

Correspondending Plain Language Summary

"Racial justice movements often have a strong presence on college campuses. Since the Black Lives Matter movement emerged in 2013, a series of protests have occurred in universities across the United States to push for fair treatment of racial minorities.

Protests typically aim to gain the attention of broader society and mobilize greater support for their cause. We can observe this from the range of tactics and contentious actions that protesters typically undertake to disrupt everyday life. Thus, being confronted by a protest might trigger backlash among some people. We therefore aimed to understand how people react when they directly encountered a protest in their social environment.

We surveyed members of a campus community exactly a week before, and during a racial justice protest that occurred at a U.S. university. The research distributed brief anonymous surveys to participants who happened to be at the location of the protest. We found that among people who had relatively weaker perceptions of campus racism, they reported more negative attitudes toward racial justice protests and protesters, as well as less support for anti-racist efforts on campus when they physically encountered a racial justice protest (compared to when they did not encounter a protest).

Although protests are a means toward social and political change, it might trigger immediate backlash among people who do not perceive a strong sense of injustice. By focusing on the attitudes of people who encounter a protest, this study contributes to our understanding of how protests can influence the attitudes of broader society." (Selvanathan & Lickel, 2019, p. 598)

Reference

Selvanathan, H. P., & Lickel, B. (2019). A field study around a racial justice protest on a college campus: The proximal impact of collective action on the social change attitudes of uninvolved bystanders. *Journal of Social and Political Psychology*, *7*(1), 598–619. https://doi.org/10.5964/jspp.v7i1.1063

1.3 Study C

Scientific abstract

"The refugee crisis in the summer of 2015 mobilized thousands of volunteers in Hungary to help refugees on their journey through Europe despite the government's hostile stance. We conducted

a survey (N = 1459) among people who were active in supporting refugees and providing services to them to test the hypothesis of whether volunteers in the context of this humanitarian crisis had social change motivations similar to those engaged in direct political activism. Hierarchical regression analysis and mediation analysis revealed the importance of opinion-based identity and moral convictions as predictors of volunteerism, while efficacy beliefs and anger only predicted political activism. Our findings suggest that volunteers engaged in helping refugees based on motivations previously described as drivers of mobilization for political activism, but chose volunteerism to alleviate the problems embedded in the intergroup situation. Although the context of the refugee crisis in Hungary may have been somewhat unique, these findings have implications for other asymmetrical politicized intergroup relations in which advantaged group members can choose to offer humanitarian aid, engage in political actions to change the situation, or do both." (Kende et al., 2017, p. 260)

Correspondending Plain Language Summary

"The refugee crisis in the summer of 2015 mobilized thousands of volunteers in Hungary to help refugees on their journey through Europe. Because of the Hungarian government's explicitly hostile stance toward refugees, offering volunteer help was treated as an expression of political dissent by authorities.

We investigated the motivations of volunteers within this political climate. The psychological motivations to engage in political protest and volunteerism can be distinguished based on previous research. Volunteerism is the intentional engagement in helping for the benefit of others; it can be long term or flare up in moments of crisis, but it does not necessarily entail intentions to bring about change. In contrast, engagement in political protest is motivated by peoples' intentions to address injustice and achieve change. As the refugee crisis evoked both types of actions (volunteerism and political protests), it provided us with the opportunity to investigate whether volunteering was driven by (1) motivation to bring about social change, (2) identification with the pro-refugee movement, and (3) experiencing a violation to their moral principles, all of which are typical for political activists.

We conducted a survey among people who were active in supporting refugees, or participated in political protests. 1459 participants completed our online survey. We measured their level of moral conviction, identification with the pro-refugee opinion group, anger about the situation, and belief in their group's efficacy to achieve change. Our results showed that identification with the pro-refugee movement and moral conviction were important motivations primarily for volunteers, while belief in the efficacy of the movement and anger were more closely related to engagement in political activism.

We therefore suggest that activities of pro-refugee volunteers became the means to express moral convictions and a desire for social change. We used the case of the refugee crisis to draw attention to the importance of understanding the similarities and differences in the paths toward volunteerism and political activism, in terms of peoples' motivation to achieve change, as social movements are just as dependent on mobilizing allies for political actions as they are on mobilizing volunteers." (Kende et al., 2017, p. 261)

Reference

Kende, A., Lantos, N. A., Belinszky, A., Csaba, S., & Lukács, Z. A. (2017). The politicized motivations of volunteers in the refugee crisis: Intergroup helping as the means to achieve social change. *Journal of Social and Political Psychology*, *5*(1), 260–281. https://doi.org/10.5964/jspp.v5i1.642

1.4 Study D

Scientific abstract

"Much of identity formation processes nowadays takes place online, indicating that intergroup differentiation may be found in online communities. This paper focuses on identity formation processes in an open online xenophobic, anti-immigrant, discussion forum. Open discussion forums provide an excellent opportunity to investigate open interactions that may reveal how identity is formed and how individual users are influenced by other users. Using computational text analysis and Linguistic Inquiry Word Count (LIWC), our results show that new users change from an individual identification to a group identification over time as indicated by a decrease in the use of 'I' and increase in the use of 'we'. The analyses also show increased use of 'they' indicating intergroup differentiation. Moreover, the linguistic style of new users became more similar to that of the overall forum over time. Further, the emotional content decreased over time. The results indicate that new users on a forum create a collective identity with the other users and adapt to them linguistically." (Bäck et al., 2018, p. 76)

Correspondending Plain Language Summary

"All humans have a fundamental desire to belong to social groups and create collective identities with groups. Groups online, such as chat forums, may also constitute a social group in a psychological sense. This means that individuals joining an online forum may start to identify with the other users, and hence adapt to these users. Moreover, users may start to distance themselves from other groups, so called outgroups. This could be problematic, for example if the forum is anti-immigrant and these other groups are immigrant groups.

This study was conducted to explore how processes of group identification and adaption can be studied in an online forum. Specifically, this is of importance if such forums are used to spread anti-immigrant messages or to recruit people to radical groups.

In order to explore how identification processes and adaption takes place in an online milieu, we have to use the text generated by the users in an online forum. Much of these processes can be studied analyzing how language is used. We use a computerized method, which basically counts the use of specific types of words, and we follow individual users over time from the time they join the forum. In the present analyses, we used text data comprised of about 60 000 000 words. The forum that we analysed in this study is an online forum in Sweden, which has over one million users. The sub-forum that we focused on is under the theme Immigration and integration. Even though the online forum is not explicitly racist, it is heavily dominated by anti-immigrant sentiments. The linguistic analysis revealed two major findings. First, we see a change in the use of pronouns over time. The use of 'I' decreases, while the use of 'we' increases when an individual has participated for a longer time in the discussions in the forum. In addition, the use of 'they' increases over time. Second, we see that the linguistic style of new users becomes more similar to the linguistic style of the forum as a whole over time, indicating that individuals adapt to the other users in the forum.

These findings indicate that processes of group identification and adaption take place in online forums in a similar way as in real life settings. The decrease in use of 'I' and increase in use of 'we' signals a collective identity formation within the forum. The increased use of 'they' signals increased distancing to one or more outgroups. The linguistic adaption is also a signal of normal group processes, showing that individuals in online forums want to be part of the group and hence adapts to the norms of the group." (Bäck et al., 2018, p. 76)

Reference

Bäck, E. A., Bäck, H., Sendén, M. G., & Sikström, S. (2018). From I to we: Group formation and linguistic adaption in an online xenophobic forum. *Journal of Social and Political Psychology, 6*(1), 76–91. https://doi.org/10.5964/jspp.v6i1.741

2 Supplementary Material B

Transcript of the Debiasing Video (English Translation)

The following transcript contains the English translation of the animated debiasing video which was presented to participants in the debiasing condition. The original debiasing video in the study was in German language and has been translated into English here to make it accessible to an international audience.

[Title]
Overconfidence in scientific topics

[Narrator]
What is the easiness effect of science popularization?

The easiness effect of science popularization (in German: Erleichterungseffekt der Wissenschaftspopularisierung) refers to the phenomenon that the simplification of scientific information influences the willingness of laypeople to rely on their own judgment rather than that of experts. Particularly in the communication of scientific information this effect is present.

The content of scientific articles is often difficult to understand for most people who are not experts – and usually in English and full of technical terms. If scientific information is presented in a simplified form, for example, technical jargon is translated into easily understandable language or complicated scientific information, such as statistical analysis or research designs, is omitted. These simplifications make scientific topics easier to understand for laypeople, which in turn enables them to participate better in the scientific discourse.

On the other hand, simplification also means that laypeople are more likely to rely on their own opinion on the scientific topic than on that of experts. As a layperson generally does not have the same competence as an expert, they overlook the limits of and overestimate their competence – which is the easiness effect.

Why do people overestimate themselves?

By formulating complex information in a way that is easy to understand, even people who are not experts understand complicated scientific topics. Understanding a complicated scientific topic makes people feel good – they have a positive emotional state. The perceived understanding and positive feelings go hand in hand with a strong perceived competence in the scientific field. If a decision is to be made on a topic that has been presented in an easily understandable way, people with a strong perceived competence tend to not consider the opinion of an expert.

What are the negative consequences of the easiness effect?

The simplification of scientific information goes hand in hand with people overestimating their ability to make decisions based on the information they understand. This information is still a simplified presentation that does not cover the entire complexity of the research field and omits technical terms and overly complex information.

After a simplified presentation of scientific information, people tend to rely on their own assessment and not consult experts or additional information. This is precisely what would be important to obtain an even more differentiated picture of a topic. After reading a simplified article, a layperson is not yet an expert and cannot judge the research field any better than those who have been studying the topic for years. Important decisions about medical treatments, the use of social media, or even whether and which vaccine to choose can be made prematurely after reading a simplified article. Scientific topics are usually complicated, complex, and rarely offer a universally valid answer, but rather a differentiated, sometimes contradictory picture.

How can you counteract the easiness effect?

The easiness effect of science popularization can certainly be counteracted. People who are not experts in the research field can become aware of the complexity of scientific topics and their own knowledge gaps. It is important to keep in mind that the presented information is simplified. Usually, the topics are more complex, more complicated and not yet fully investigated. Consulting further information and/or asking an expert are good ways to avoid overestimating your own competence and to make appropriate decisions.

3 Supplementary Tables

3.1 **Supplementary Table A.** Factor analysis of intended social media reactions, comparing principal component analysis (PCA) and principal axis factoring (PAF).

	PCA	PAF
	Factor loadings	Factor loadings
Items		
Intended social media reactions		
1. Liking	0.771	0.665
2. Disliking	0.641	0.496
3. Sharing	0.806	0.725
4. Comment on	0.852	0.827
Total explained variance	0.595	0.474
KMO	0.738	0.738
Bartlett's test	< 0.001	< 0.001

Note. One component was extracted with eigenvalues ≥ 1, so that no rotation was performed.

3.2 Supplementary Table B. Effect of abstract video type on intended consumer reactions (aggregated and individual).

Type of intended reaction	Video abstract type				*t*	*p*	Hedge's *g*
	Animated PLS		Animated scientific abstracts				
	M	*SD*	*M*	*SD*			
Knowledge-enhancing reactions (α = 0.86)	4.18	1.31	4.02	1.22	0.87	0.388	0.13
Reading comments related to the video abstract	4.84	1.55	4.58	1.33	1.21	0.228	0.18
Watching another video of the study	3.98	1.54	4.00	1.45	–0.09	0.925	–0.01
Watching another video of the same topic	4.35	1.51	4.26	1.39	0.38	0.703	0.06
Getting full access to the paper in German	3.98	1.78	3.74	1.65	0.92	0.361	0.14
Getting full access to the paper in English	3.77	1.79	3.50	1.78	0.98	0.328	0.15
Social media reactions (α = 0.77)	2.78	1.29	2.64	1.12	0.77	0.441	0.12
Liking	3.37	1.76	3.42	1.58	–0.23	0.822	–0.03
Sharing	2.86	1.47	2.53	1.33	1.59	0.114	0.24
Commenting	2.11	1.39	1.97	1.08	0.74	0.462	0.11

3.3 **Supplementary Table C.** Intercorrelation matrix of dependent variables.

Variable	1	2	3	4	5
1. Comprehensibility					
2. Credibility	$.537^{***}$				
3. Confidence in ability to evaluate the study	$.207^{**}$	$.516^{***}$			
4. Ability to make decisions without further information	.106	$.353^{***}$	$.724^{***}$		
5. Knowledge-enhancing reactions	$.355^{***}$	$.152^{*}$	.119	-.014	
6. Social media reactions	$.202^{**}$	$.334^{***}$	$.453^{***}$	$.439^{***}$	$.395^{***}$

Note. $n = 179$. * indicates $p < 0.05$. ** indicates $p < 0.01$. *** indicates $p < 0.001$.
